# Human adipose-derived mesenchymal stem cells prevent type 1 diabetes induced by immune checkpoint blockade

**DOI:** 10.1007/s00125-022-05708-3

**Published:** 2022-05-05

**Authors:** Emi Kawada-Horitani, Shunbun Kita, Tomonori Okita, Yuto Nakamura, Hiroyuki Nishida, Yoichi Honma, Shiro Fukuda, Yuri Tsugawa-Shimizu, Junji Kozawa, Takaaki Sakaue, Yusuke Kawachi, Yuya Fujishima, Hitoshi Nishizawa, Miyuki Azuma, Norikazu Maeda, Iichiro Shimomura

**Affiliations:** 1 Department of Metabolic Medicine, Graduate School of Medicine, Osaka University, Osaka, Japan; 2 Department of Adipose Management, Graduate School of Medicine, Osaka University, Osaka, Japan; 3 ROHTO Pharmaceutical Co., Ltd, Osaka, Japan; 4 Department of Diabetes Care Medicine, Graduate School of Medicine, Osaka University, Osaka, Japan; 5 Department of Molecular Immunology, Graduate School of Medical and Dental Sciences, Tokyo Medical and Dental University, Tokyo, Japan; 6 Department of Metabolism and Atherosclerosis, Graduate School of Medicine, Osaka University, Osaka, Japan

**Keywords:** Immune checkpoint inhibitor, Mesenchymal stem cells, NOD mouse

## Abstract

**Aims/hypothesis:**

Immunomodulators blocking cytotoxic T-lymphocyte-associated protein 4 (CTLA-4) and programmed cell death protein 1 (PD-1) or programmed death-ligand 1 (PD-L1) have improved the treatment of a broad spectrum of cancers. These immune checkpoint inhibitors (ICIs) reactivate the immune system against tumour cells but can also trigger autoimmune side effects, including type 1 diabetes. Mesenchymal stem cell (MSC) therapy is the most prevalent cell therapy, with tissue-regenerating, anti-fibrosis and immunomodulatory functions provided by the secretome of the cells. Here, we examined whether systemic MSC treatment could prevent the development of type 1 diabetes in a NOD mouse model.

**Methods:**

The purified PD-L1 monoclonal antibody was administered to induce diabetes in male NOD mice which normally do not develop diabetes. Human adipose-derived MSCs were administered by tail vein injections. T cells, macrophages and monocyte-derived macrophages expressing C-X-C motif chemokine ligand 9 (CXCL9) in pancreatic sections of NOD mice and a cancer patient who developed diabetes following the ICI treatments were analysed by immunofluorescence. Tissue localisation of the injected MSCs, plasma exosome levels and plasma cytokine profiles were also investigated.

**Results:**

PD-1/PD-L1 blockade induced diabetes in 16 of 25 (64%) NOD mice which received anti-PD-L1 mAb without hMSCs [MSC(−)], whereas MSC administration decreased the incidence to four of 21 (19%) NOD mice which received anti-PD-L1 mAb and hMSCs [MSC(+)]. The PD-1/PD-L1 blockade significantly increased the area of CD3-positive T cells (6.2-fold) and macrophage-2 (Mac-2) antigen (2.5-fold)- and CXCL9 (40.3-fold)-positive macrophages in the islets. MSCs significantly reduced T cell (45%) and CXCL9-positive macrophage (67%) accumulation in the islets and the occurrence of diabetes. The insulin content (1.9-fold) and islet beta cell area (2.7-fold) were also improved by MSCs. T cells and CXCL9-positive macrophages infiltrated into the intricate gaps between the beta cells in the islets by PD-1/PD-L1 blockade. Such immune cell infiltration was largely prevented by MSCs. The most striking difference was observed in the CXCL9-positive macrophages, which normally did not reside in the beta cell region in the islets but abundantly accumulated in this area after PD-1/PD-L1 blockade and were prevented by MSCs. The CXCL9-positive macrophages were also observed in the islets of a cancer patient who developed diabetes following the administration of ICIs but few CXCL9-positive macrophages were observed in a control patient. Mechanistically, the injected MSCs accumulated in the lung but not in the pancreas and strongly increased plasma exosome levels and changed plasma cytokine profiles.

**Conclusions/interpretation:**

Our results suggest that MSCs can prevent the incidence of diabetes associated with immune checkpoint cancer therapy and may be worth further consideration for new adjuvant cell therapy.

**Graphical abstract:**

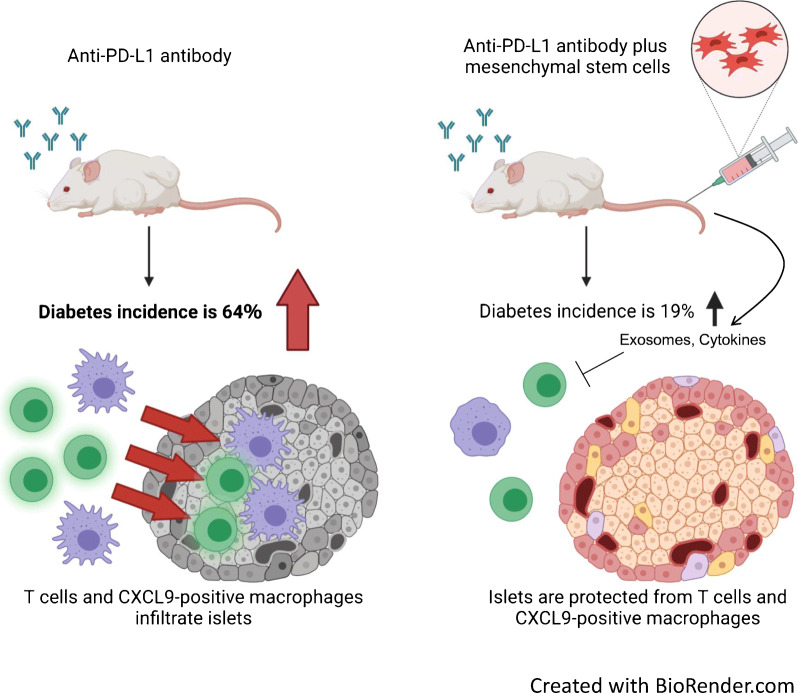

**Supplementary Information:**

The online version of this article (10.1007/s00125-022-05708-3) contains peer-reviewed but unedited supplementary material..

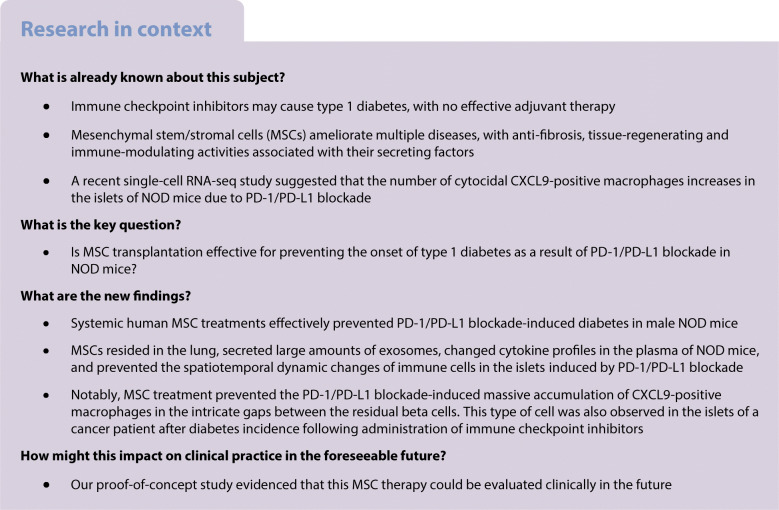



## Introduction

Most incipient tumours can be eliminated by immune surveillance, while tumour cells that can evade this immune system response develop into cancer. One tumour cells use for immune surveillance evasion is the upregulation of immune checkpoint expression, such as that of programmed death-ligand 1 (PD-L1), which attenuates T cell activation through the counterpart receptor programmed cell death protein 1 (PD-1) expressed on the surface of T cells. Inhibiting immune checkpoint signalling, such as by PD-1/PD-L1 ligation with neutralising antibodies, can restore T cell activation, allowing T cells to engage in tumour cell killing [1].

Immune checkpoint inhibitors (ICIs) have increasingly achieved great success in the treatment of several malignancies. Therapeutic targeting of these pathways can lead to imbalances in immune tolerance, which manifest as immune-related adverse events, including type 1 diabetes [2]. ICI-associated type 1 diabetes is one of the immune-related side effects, with a variable prevalence between 0.2% and 1.4% [3–7]. The course of disease progression varies from typical fulminant type 1 diabetes to acute-onset type 1 diabetes [8]. In many cases, it gradually progresses for months to completely lose beta cells [8], contrasting with the immediate progression of the fulminant type 1 diabetes within a few days [9]. The preservation of residual beta cells is thought to be important for metabolic wellbeing and the prevention of complications in type 1 diabetes [10].

PD-1 is expressed by T cells activated by the T cell receptor signalling pathway [11–13], whereas PD-L1 is expressed by islet parenchymal cells and antigen-presenting cells, such as macrophages and dendritic cells [14, 15]. Inhibition of the PD-1 pathway in diabetes-susceptible NOD mice, an animal model of nonobese spontaneous type 1 diabetes, was reported to accelerate the onset of type 1 diabetes [16]. Mice deficient in PD-1 were reported to develop autoimmune diseases, including type 1 diabetes, depending on their genetic backgrounds [17].

Mesenchymal stem cells (MSCs), also referred to as mesenchymal stromal cells, have attracted attention for their ability to modulate immune function in addition to regulating fibrosis and tissue regeneration [18]. The secretome of MSCs, including cytokines and exosomes, is considered to play an important therapeutic role in addition to the differentiation of MSCs themselves, which compensates for damaged tissues [19]. We previously demonstrated that exosome secretion played a fundamental role in the use of MSCs to treat a pressure overload-induced heart failure model [20].

The therapeutic application of cultured MSCs was found to be effective for both type 1 and type 2 diabetes [21, 22]. It was also reported that MSCs partially protected female NOD mice from developing spontaneous diabetes [23]. The present study tested whether systemically administered MSCs protect male NOD mice from ICI-induced type 1 diabetes.

## Methods

### Cell transplantation

Adipose-derived human mesenchymal stem cells (hMSCs) (Lonza Bioscience, Switzerland) were cultured in Mesenchymal Stem Cell Growth Medium 2 (PromoCell, Germany) and the cells at passage 4 were freshly prepared before each experiment. The cells were mixed with DMEM low glucose + penicillin-streptomycin (P/S) at 1.0 × 10^6^ cells/100 μl and injected using a 27G needle through the tail vein at 2- to 3-day intervals for 2 weeks. In each session, the cells were slowly infused over at least 5 s. For PKH-labelled hMSC transplantation experiments, hMSCs were labelled with a PKH67 green fluorescent dye (Merck, Germany) according to the protocol supplied by the manufacturer.

### Animals

Male NOD/Shi_Jcl_ mice were purchased from CLEA Japan (Japan). Mice were housed in cages in a room at 22°C under a 12 h/12 h light/dark cycle (lights off from 08:00 hours to 20:00 hours). Mice were randomly allocated to the control, anti-PD-L1 antibody [MSC(−) group] or anti-PD-L1 antibody plus MSC group [MSC(+) group] so that blood glucose and body weight means at the start of the experiment were even. Blinding was not carried out in any of the experiments.

### Human pancreas specimens

We obtained pancreas specimens from a 65-year-old male patient (whose BMI was 19.7 kg/m^2^) who developed type 1 diabetes after the administration of ICIs [24]. At the age of 60, he was diagnosed with type 2 diabetes. Under the administration of vildagliptin, his HbA_1c_ was maintained under 41 mmol/mol (6%). Two years later, he was diagnosed with pancreas metastasis of renal carcinoma, and cytotoxic T-lymphocyte-associated protein 4 (CTLA-4) antibody (ipilimumab) and anti-PD-1 antibody (nivolumab) combination therapy was started. Ipilimumab and nivolumab were administered four times over 2 months and ten times over 6 months, respectively. Six months after the start of administration, he developed diabetic ketoacidosis and his serum C-peptide was less than the measurement sensitivity. Thereafter, the combination therapy was discontinued. Two years after developing diabetic ketoacidosis, total pancreatectomy was performed for the radical treatment of carcinoma. Histochemistry was performed in the nontumour region of the pancreas from the patient.

As a control, we also evaluated the pancreas specimen of a 70-year-old male individual whose BMI was 24.2 kg/m^2^ with normal glucose tolerance who had undergone pancreatic resection [24].

### Anti-PD-L1 monoclonal antibody

The hybridoma cells (MIH5) producing the anti-PD-L1 monoclonal antibody were cultured [25].

The anti-PD-L1 monoclonal antibody was purified from the culture supernatant of MIH5 hybridoma cells and concentrated to 5 mg/ml and intraperitoneally administered to the mice using a 27G needle; 1000 μg on day 0, followed by 500 μg on days 2, 5, 7, 9 and 12.

### Measurement of blood glucose

Blood glucose was measured from the tail vein on days 0, 2, 5, 7, 9, 12 and 14 in the ad lib-fed state and assayed using Glutest Neo alpha (Sanwa Kagaku Kenkyusho, Japan). Mice with blood glucose levels greater than 13.9 mmol/l were regarded as diabetic [16]. Those with levels exceeding the upper limit of blood glucose measurement were recorded as 33.3 mmol/l.

### Histochemistry

Mouse pancreas samples were dissected, fixed in 4% paraformaldehyde in PBS at 4°C and embedded in paraffin. Human pancreases were also embedded in paraffin after pancreatectomy. In the pancreas from the case patient, the only nontumour region was used for paraffin blocks. The sections (2 μm thick) were deparaffinised and stained with H&E. The deparaffinised sections were also treated with citrate buffer (TRS pH 6.0, Agilent, USA) using a high-pressure oven for 30 s at 121°C and 10 s at 90°C. The sections were blocked for 30 min at room temperature using a blocking buffer consisting of 3% BSA. The sections stained for insulin (guinea pig anti-insulin, IR002; Agilent) were visualised with anti-guinea pig horseradish peroxidase (HRP)-conjugated antibody (A-18775; Thermo Fisher Scientific, USA) and 3,3′-diaminobenzidine tetrahydrochloride (DAB) to evaluate the residual islet area. For the quantification of the islet area, the area was visualised and analysed using a BZ-X700 microscope with built-in software (Keyence, USA). Other sections used for fluorescence staining were analysed by a FLUOVIEW FV3000 laser scanning confocal microscope (Olympus, Japan) for micro-imaging or by a BZ-X700 microscope (Keyence) for macro-imaging.

The following primary antibodies were diluted with Dulbecco’s PBS without calcium and magnesium [PBS(−)] containing 1% bovine serum albumin (BSA) and used: rabbit anti-mouse and human CD3 (1:2, IR503; Agilent); rabbit anti-mouse CD4 (1:100, ab183685; Abcam, UK); rabbit anti-mouse CD8 (1:100, ab217344; Abcam); rat anti-mouse and human macrophage-2 (Mac-2) antigen (1:100, CL8942AP; Cedarlane, Canada); goat anti-mouse C-X-C motif chemokine ligand 9 (CXCL9) (1:100, AF-492-NA; R&D Systems, USA); rabbit anti-human CXCL9 (1:50, ab202961; Abcam); rabbit anti-human, mouse and rat PD-L1 (1:100, 17952-1-AP; Proteintech, USA); and mouse anti-human glucagon (1:1000, 22160318; Merck). The following secondary antibodies were diluted with PBS(–) containing 1% BSA and used: Alexa Fluor 594 donkey anti-guinea pig IgG (1:400, 705-585-148; Jackson ImmunoResearch, USA); Alexa Fluor 647 donkey anti-guinea pig IgG (1:400, 106-605-003; Jackson ImmunoResearch); Alexa Fluor 594 goat anti-mouse IgG (1:400, A-11005; Thermo Fisher Scientific); biotin-conjugated donkey anti-rabbit IgG (1:200, 711-066-152; Jackson ImmunoResearch); biotin-conjugated donkey anti-rat IgG (1:200, 712-066-153; Jackson ImmunoResearch); biotin-conjugated rabbit anti-rat IgG (1:200, BA-4000; Vector Laboratories, USA); biotin-conjugated rabbit anti-goat IgG (1:200, PK-6105; Vector Laboratories). For biotinylated antibodies, streptavidin conjugated to Alexa Flour 488 (1:200, S32354; Thermo Fisher Scientific) or streptavidin conjugated to Alexa Flour 647 (1:200, S21374; Thermo Fisher Scientific) were used. The characteristics and validation of the antibodies are listed on the manufacturer’s site. In quadruple immunofluorescence staining for DAPI, insulin, CD3 and Mac-2 antigen or CXCL9 in NOD mouse islets, insulin and Mac-2 antigen or CXCL9 were stained simultaneously, and CD3 and DAPI were stained sequentially. In triple immunofluorescence staining for insulin, glucagon and CD3 or CXCL9 in human islets, they were stained simultaneously.

### Measurement of insulin content

We extracted pancreatic insulin by acid-ethanol, measured insulin content by a Morinaga Ultra-Sensitive Mouse Insulin ELISA kit (Morinaga, Japan) according to the protocol supplied by the manufacturer and calculated the insulin content/protein content (pmol/mg).

### Measurement of immune cell-positive areas

The pancreatic specimens of the three mice with insulin contents close to the mean value in each group were selected as the group representatives. Following staining with anti-insulin, CD3, CD4, CD8, Mac-2 antigen and CXCL9 antibodies, five pancreatic islets in each section of the group representatives were randomly sampled from each mouse, and 15 islets in each group were analysed. The islet sections were analysed using a FLUOVIEW FV3000 laser scanning confocal microscope (Olympus), and the cell occupancy of each immune cell-positive area in each islet area was calculated with ImageJ software (version 1.53f51; National Institutes of Health, USA).

### Exosome isolation

The plasma sample was mixed with thrombin (500 U/ml) for 10 min followed by centrifugation at 12,000 *g* for 20 min to remove fibrin. For exosome isolation, the defibrinated plasma was mixed with ExoQuick (System Biosciences, USA) for 30 min on ice and centrifuged for 30 min at 1500 *g*. The resultant pellet was dissolved overnight with PBS(−) and ultracentrifuged at a mean of 110,000 *g* for 2 h, followed by a washing step of the exosome pellet with PBS(−) at a mean of 110,000 *g* for 2 h (S80AT2 rotor, Eppendorf, Japan).

### Western blotting

The isolated exosome pellets were dissolved in an SDS–sample buffer and the proteins were separated by a 4–20% gradient SDS–PAGE gel (Bio-Rad, USA) and transferred onto a nitrocellulose membrane. The membranes were blocked with Block-One blocking reagent (Nakarai Tesque, Japan) and then incubated with primary antibodies dissolved in Can Get Signal Solution 1 (TOYOBO, Japan) overnight at 4°C, followed by incubation with secondary antibodies conjugated with HRP using Can Get Signal Solution 2 (TOYOBO) for 60 min at room temperature. Chemiluminescence signals developed with Chemi-Lumi One Super (Nakarai Tesque) were visualised by ChemiDoc Touch (Bio-Rad) and quantified using Image Lab software (version 6.0.1; Bio-Rad). The following primary antibodies were used: mouse monoclonal anti-Alix (sc53538; Santa Cruz Biotechnology, USA); mouse monoclonal anti-human CD63 (BD556019; BD Biosciences, USA); sheep polyclonal anti-human milk fat globule-EGF factor 8 protein (MFG-E8) (AF2767; R&D Systems); and rabbit polyclonal anti-syntenin (ab19903; Abcam). The following secondary antibodies were used: HRP-conjugated rabbit anti-sheep IgG (Thermo Fisher Scientific); HRP-conjugated sheep anti-mouse IgG (GE Healthcare, USA); and HRP-conjugated donkey anti-rabbit IgG (GE Healthcare).

### Cytokine array

The plasma samples of the mice in each group were pooled, and 0.5 ml of the pooled plasma was analysed by using the Cytokine Array–Human Cytokine Antibody Array (Membrane, 4 Targets, Abcam) according to the protocol supplied by the manufacturer. Chemiluminescence signals were visualised by ChemiDoc Touch (Bio-Rad) and quantified using Image Lab software (version 6.0.1; Bio-Rad).

### Ethical considerations

The experimental protocol was approved by the Ethics Review Committee for Animal Experiment of the Osaka University School of Medicine. This study also conforms to the Guide for the Care and Use of Laboratory Animals published by the US National Institutes of Health. Both patients whose pancreas tissues were used in this study provided written informed consent before their participation. This study was approved by the Human Ethics Committee of Osaka University (no. 17459-4) and was carried out in accordance with the Declaration of Helsinki.

### Statistical analysis

**Fig. 1:**
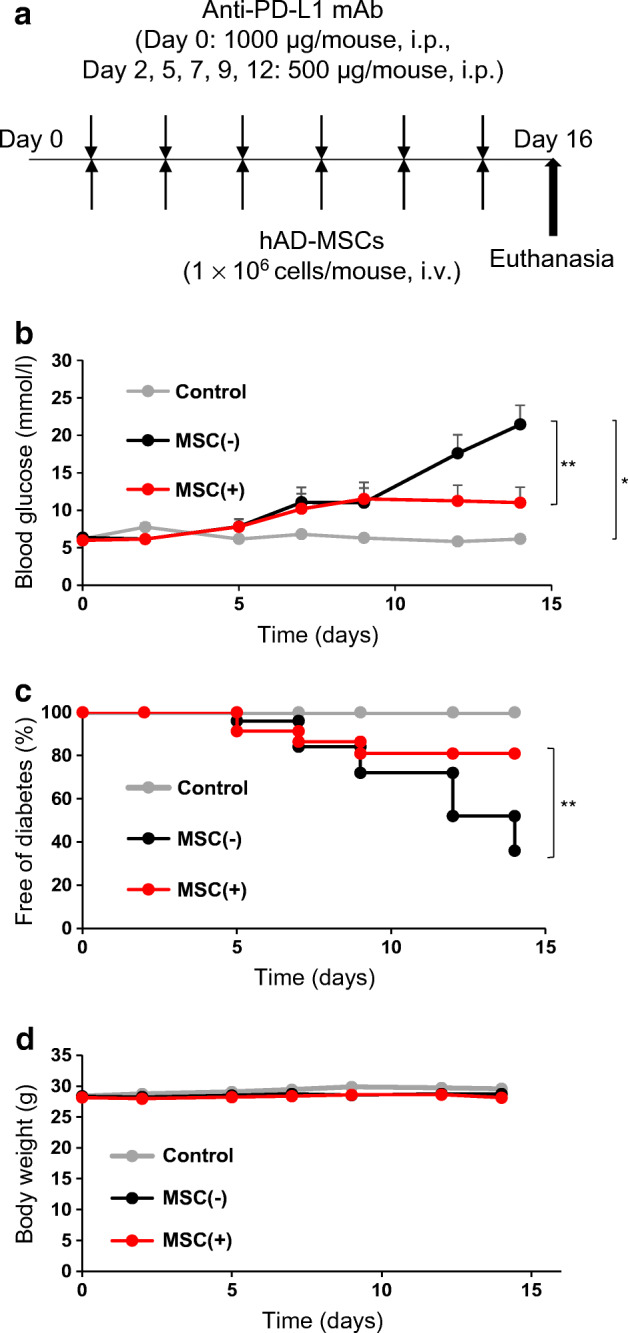
Suppressive effect of hMSCs on type 1 diabetes induced by an anti-PD-L1 monoclonal antibody (mAb). (**a**) Experimental design for the intraperitoneal injection of anti-PD-L1 mAb and intravenous injection of hMSCs in NOD mice. Anti-PD-L1 mAb was injected intraperitoneally; 1000 μg on day 0, followed by 500 μg on days 2, 5, 7, 9 and 12. hMSCs were injected at 1.0 × 10^6^ cells per mouse via the tail vein and injections were performed on the same days as the injections of anti-PD-L1 mAb. (**b**) The mean blood glucose level in each group. Blood glucose levels were measured with a simple glucose meter. Those exceeding the upper limit of measurement with a simple blood glucose meter were regarded as having a blood glucose level of 33.3 mmol/l. (**c**) Diabetes-free rate after the injection of anti-PD-L1 mAb. Mice with blood glucose levels greater than 13.9 mmol/l were regarded as diabetic. (**d**) Mean body weight in each group. (**b**, **c**, **d**) Control mice (not receiving anti-PD-L1 mAb or hMSCs), *n*=3; MSC(−) mice, *n*=25; MSC(+) mice, *n*=23. Data are shown as the mean ± SEM. *p˂0.05; **p˂0.01 between the indicated two groups, at day 14 by Student’s *t* test (**b**, **d**) and logrank test (**c**). hAD-MSCs, human adipose-derived mesenchymal stem cells

**Fig. 2:**
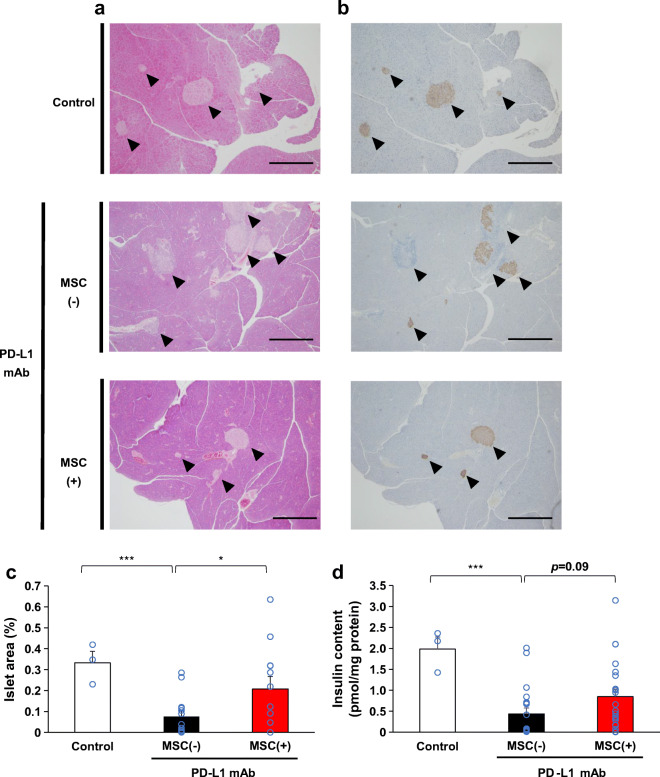
Morphological changes and residual pancreatic beta cells. (**a**) Representative H&E staining and (**b**) anti-insulin immunostaining of the pancreas 16 days after initiating anti-PD-L1 monoclonal antibody (mAb) treatments: pancreas from a mouse that did not receive anti-PD-L1 mAb or hMSCs (control: normoglycaemic, insulin content was 2.17 pmol/mg), pancreas from an MSC(–) mouse (normoglycaemic, insulin content was 0.41 pmol/mg) and pancreas from an MSC(+) mouse (normoglycaemic, insulin content was 0.95 pmol/mg). The arrowheads indicate islets. Scale bars, 400 μm. (**c**) The islet area (%) in a whole pancreatic section between groups [control, *n*=3; MSC(−), *n*=14; MSC(+), *n*=12]. (**d**) Comparison of insulin content in the pancreas between groups [control, *n*=3; MSC(−), *n*=20; MSC(+), *n*=21]. Data are shown as the mean ± SEM. *p˂0.05; ***p˂0.001 between the indicated two groups by Student’s *t* test

**Fig. 3:**
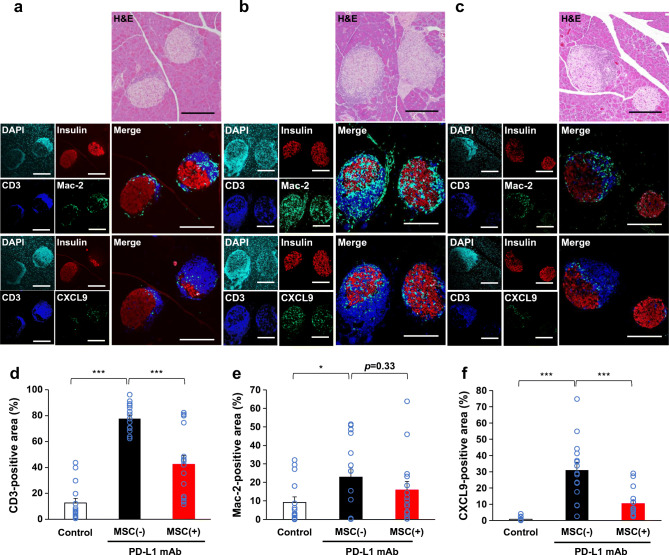
Changes of immune cell profiles of the islets. Representative immunostaining images of the pancreases of mice in the three groups (**a**–**c**). (**a**) Pancreas from a mouse not receiving anti-PD-L1 monoclonal antibody (mAb) or hMSCs (control: normoglycaemic, insulin content was 2.17 pmol/mg). (**b**) Pancreas from an MSC(−) mouse (normoglycaemic, insulin content was 0.41 pmol/mg). (**c**) Pancreas from an MSC(+) mouse (normoglycaemic, insulin content was 1.00 pmol/mg). (**a**–**c**) Upper panels: H&E staining. Middle panels: quadruple immunofluorescence staining for DAPI (light blue), insulin (red), CD3 (blue) and Mac-2 antigen (green), and their merge. Lower panels: quadruple immunofluorescence staining for DAPI, insulin, CD3 and CXCL9 (green), and their merge. The upper to lower panels are 2 μm serial sections. Scale bars, 200 μm. (**d**–**f**) Comparison of the immunostaining-positive area of the pancreas between groups. (**d**) CD3-positive area. (**e**) Mac-2 antigen-positive area. (**f**) CXCL9-positive area [control group islets: *n*=15; MSC(−) group islets: *n*=15; MSC(+) group islets: *n*=15]. Data are shown as the mean ± SEM. *p˂0.05; ***p˂0.001 between the indicated two groups by Student’s *t* test

**Fig. 4:**
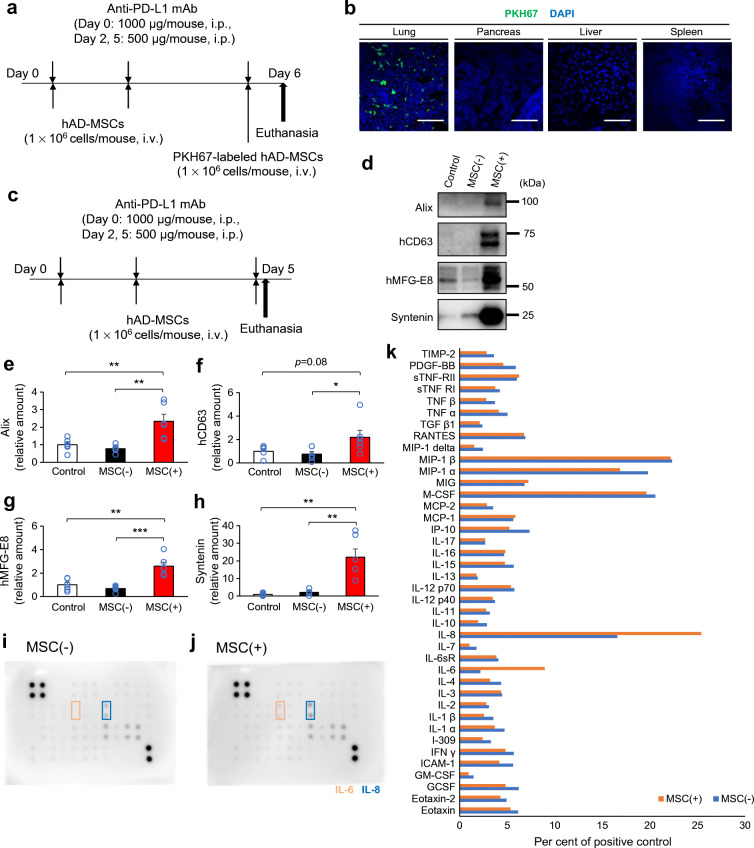
Possible secretome-mediated immunomodulation by MSCs. (**a**) Experimental design for the intraperitoneal injection of anti-PD-L1 monoclonal antibody (mAb) and intravenous injection of PKH-labelled hMSCs in NOD mice. The tissues were dissected 24 h after the final injection. (**b**) Fluorescence of PKH and DAPI in the lung, pancreas, liver and spleen. Scale bars, 100 μm. (**c**) Experimental design for the intraperitoneal injection of anti-PD-L1 mAb and intravenous injection of hMSCs in NOD mice. The blood plasma was collected at 4 h after final injections [control: mean blood glucose level was 7.5 mmol/l; MSC(−): mean blood glucose level was 11.6 mmol/l; MSC(+): mean blood glucose level was 7.6 mmol/l; those exceeding the upper limit of measurement with a simple blood glucose meter were regarded as having a blood glucose level of 33.3 mmol/l]. (**d**–**h**) Plasma extracellular vesicles (EVs) were purified, and the amounts of exosome marker proteins were evaluated by western blotting. Exosome markers: Alix and syntenin by human and mouse species unspecific antibodies; CD63 and MFG-E8 by human-specific antibodies (**d**). (**e**–**h**) Comparison of the plasma exosome levels between groups by exosome markers in EV-fractions: Alix (**e**), hCD63 (**f**), hMFG-E8 (**g**) and syntenin (**h**) (all groups *n*=6). (**e**–**h**) Data are shown as the mean ± SEM. *p˂0.05; **p<0.01; ***p˂0.001 between the indicated two groups by Student’s t test. (**i**–**k**) Plasma cytokine profile was determined in cytokine arrays using pooled plasma (*n*=6) of the MSC(−) group (**i**) and the MSC(+) group (**j**). Cytokine profiles were compared between groups (**k**). hAD-MSCs, human adipose-derived mesenchymal stem cells; hCD63, human CD63; hMFG-E8, human milk fat globule-EGF factor 8 protein

The data are expressed as the mean ± SEM. Differences between the indicated two groups were analysed by two-sided Student’s *t* test (Figs. 1b, d, 2c, d, 3d, e, f, 4e–h). For Kaplan–Meier analysis (Fig. 1c), logrank test was performed between the indicated two groups. *p* values ˂0.05 were considered statistically significant.

## Results

### Reduction in diabetes incidence as a result of hMSC treatment

A purified anti-PD-L1 monoclonal antibody was injected into male NOD mice which are known not to spontaneously develop diabetes (Fig. 1a) [16]. Six consecutive injections of anti-PD-L1 antibody for 2 weeks induced male NOD mice to develop overt diabetes and blood glucose levels greater than 13.9 mmol/l, while NOD mice without anti-PD-L1 antibody did not develop diabetes (Fig. 1b, c). To determine the effect of systemic hMSC administration, hMSCs (1 × 10^6^ cells/mouse) or serum-free medium was intravenously administered after each antibody injection. As shown in Fig. 1b, c, anti-PD-L1 antibody-induced diabetes in 16 of 25 (64%) NOD mice [MSC(−) group], whereas systemic hMSC administration decreased the incidence of overt diabetes to four of 21 (19%) and resulted in significantly lower mean blood glucose levels (Fig. 1b). The administration of hMSCs significantly suppressed the onset of diabetes [MSC(−) vs MSC(+) group, *p*=0.0065] (Fig. 1c). There was no significant difference in body weight among the three groups during the course of the study (Fig. 1d).

### Preservation of islet morphology and insulin contents

Next, we analysed the histology of the pancreas on day 16. H&E staining and immunostaining of insulin revealed typical round islets in the control group. In contrast, deformed and partially missing islets were frequently observed in the MSC(−) group that received anti-PD-L1 antibody (Fig. 2a, b), whereas in the MSC(+) group, most islets maintained their morphology and insulin positivity (Fig. 2a, b). When the residual islet beta cell area in the pancreatic section was quantified, a significantly larger beta cell area was observed in the MSC(+) group than in the MSC(−) group [2.7-fold, MSC(−): 0.076 ± 0.026%; MSC(+):0.208 ± 0.059%, *p*=0.040] (Fig. 2c). Moreover, the MSC(+) group had a non-significantly higher insulin content than the MSC(−) group [1.9-fold, MSC(−): 0.44 ± 0.14; MSC(+): 0.85 ± 0.19 pmol/mg protein, *p*=0.09] (Fig. 2d).

### Changes of the islet cellularity and localisation

PD-1/PD-L1 blockade in NOD mice was reported to facilitate diabetes progression through T cell activation and the subsequent recruitment of monocyte-derived macrophages [26]. CXCL9-positive macrophages acquire cytocidal capabilities in response to T cell-derived IFN-γ [26]. We visualised T cells (CD3-positive), macrophages (Mac-2 antigen-positive) and CXCL9-positive macrophages along with beta cells (insulin-positive) in the islets by immunofluorescence staining (Fig. 3). Representative immunofluorescence images were taken from the three mice per group that had pancreatic insulin contents close to the mean value of each group, and a total of 15 islets per group were examined and their CD3-positive T cell, Mac-2 antigen-positive macrophage and CXCL9-positive macrophage contents were quantified (Fig. 3d–f and electronic supplementary material [ESM] Fig. 1). As shown in the representative images in Fig. 3a, T cells were found to surround the islets even in control NOD mice. There was an occasional massive accumulation of T cells surrounding the beta cells even in the control mice that did not develop overt diabetes (Fig. 3a [CD3]). Mac-2 antigen-positive macrophages accumulated mainly in a similar region and were closely associated with T cells (Fig. 3a [Mac-2 antigen]). Interestingly, there were few CXCL9-positive macrophages localised in the beta cell region in the islets (Fig. 3a [CXCL9]), in contrast to the localisation of macrophages stained with Mac-2 antigen (Fig. 3a [Mac-2]). Regarding the immune cell-positive areas in the islets, few CXCL9-positive macrophages resided in the islets of the control group male NOD mice (Fig. 3f), whereas T cells (Fig. 3d) and macrophages positive for Mac-2 antigen (Fig. 3e) were observed. PD-1 blockade decreased the beta cell area (Fig. 3b) and induced the accumulation of T cells (6.2-fold) in the islets of the MSC(−) group (Fig. 3b [CD3] and Fig. 3d). The remaining islet beta cell regions were deformed and invaded by these T cells (Fig. 3b [CD3]). There was also an increase in the area of Mac-2 antigen-positive macrophages (2.5-fold) throughout the whole islet (Fig. 3b [Mac-2 antigen] and Fig. 3e). Interestingly, the area of CXCL9-positive macrophages dramatically increased (40.3-fold) (Fig. 3f) and the cells were mainly localised within the intricate gaps of the remaining beta cells (Fig. 3b [CXCL9]). MSC treatments significantly improved the beta cell area (Fig. 3c) and significantly decreased the areas of T cells (45%) and CXCL9-positive macrophages (67%) (Fig. 3d, f), while the area of Mac-2 antigen-positive macrophages was slightly decreased in the islets (Fig. 3e). Representative images of the MSC(+) group indicated that MSC treatment decreased both CD3-positive T cells and Mac-2 antigen-positive macrophages in the islets, and few T cells (Fig. 3c [CD3]) and macrophages (Fig. 3c [Mac-2 antigen]) remained in the beta cell region. The localisation of CXCL9-positive macrophages changed more clearly to the outside of the beta cell region (Fig. 3c [CXCL9]) and their positive area was significantly decreased in the islets of the MSC(+) group to less than 66% (Fig. 3f). This distinct cellular localisation difference of CXCL9-positive macrophages between the MSC(−) group and the MSC(+) group was consistently observed in a series of representative images (ESM Fig. 2). The characteristic phenomenon observed in the MSC(+) group was that T cells and CXCL9-positive macrophages were prevented from accumulating in the beta cell clusters in the islets (Fig. 3c); otherwise, these cells abundantly accumulated in the intricate gaps between the residual beta cells in the islets as a result of PD-1/PD-L1 blockade [MSC(−) group] (Fig. 3b).

We also visualised CD4-positive and CD8-positive T cells along with beta cells (ESM Fig. 3a-c). Both classes of T cells were present in the islets even in control NOD mice (ESM Fig. 3a) and were significantly increased by the PD-1/PD-L1 blockade (ESM Fig. 3b) and significantly decreased by MSC treatments (ESM Fig. 3c-e). The ratio of each class of T cells was not largely changed by this immune activation or MSC treatments (ESM Fig. 3f).

We previously reported PD-L1 expression in pancreatic beta cells of control patients but not in diabetes patients receiving ICIs [24]. The beta cell immunoreactivity for PD-L1 was positive in control NOD mice as reported previously (ESM Fig. 4a) [16] but was strongly reduced in the NOD mice treated with anti-PD-L1 antibody (ESM Fig. 4b), even though the mice used were not diabetic. Interestingly, MSC treatments prevented such reduction of PD-L1 immunoreactivity of beta cells (ESM Fig. 4c).

### The possible importance of secreted factors from the injected hMSCs

Next, we investigated the tissue localisation of the injected MSCs (Fig. 4a, b). The PKH-labelled MSCs massively localised in the lung tissues, consistent with our previous report [20]. However, there were few or no visible PKH-labelled cells in the pancreas, the spleen and the liver. MSCs are thought to function in distant organs by secreting factors such as cytokines and exosomes [19]. We evaluated both plasma exosome levels and cytokine profiles 4 h after the final MSC injection (Fig. 4c). The plasma exosome levels were evaluated by exosome marker protein levels in quantitatively purified exosome fractions from a defined plasma volume (Fig. 4d–h). The exosome markers CD63 and MFG-E8 were stained by human-specific antibodies, and therefore represented the presence of MSC-derived exosomes in plasma. Interestingly, the exosome markers Alix and syntenin were specific for both mice and humans and therefore may indicate a significant increase of plasma exosome levels by MSC injections (Fig. 4d–h). The plasma cytokines were evaluated by a cytokine array for human cytokines using pooled plasma of six mice in each group (Fig. 4i–k). MSCs increased IL-6 and IL-8, both of which MSCs were reported to secrete, while they did not increase TGF-β which was also reported to be secreted [27, 28] (Fig. 4i–k).

### Presence of T cells and CXCL9-positive macrophages in human islets

**Fig. 5:**
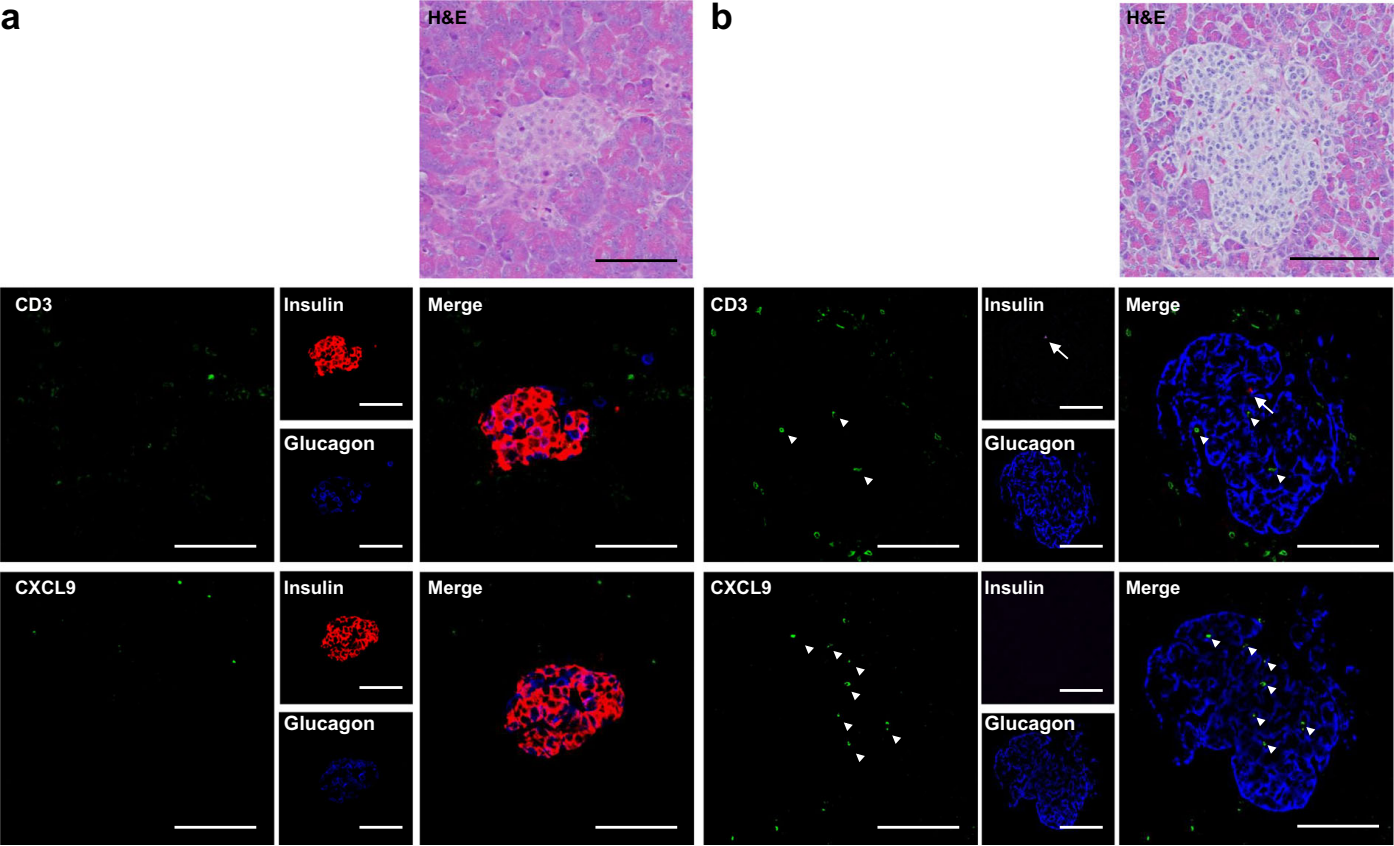
Representative immunostaining images of the pancreases of a control patient and the patient who developed type 1 diabetes after administration of ICIs. (**a**) Pancreas from a control patient. (**b**) Pancreas from the patient who developed type 1 diabetes after administration of ICIs. (**a**, **b**) Upper panels: H&E staining. Middle panels: triple immunofluorescence staining for CD3 (green), insulin (red) and glucagon (blue), and their merge. Lower panels: triple immunofluorescence staining for CXCL9 (green), insulin, and glucagon, and their merge. The upper to lower panels are 2 μm serial sections. The arrows indicate insulin and the arrowheads indicate CD3 or CXCL9. Scale bars, 100 μm

Although T cells were reported to accumulate in the islets of human patients, CXCL9-positive macrophages have not been tested in the pancreas specimens of human patients who developed diabetes after receiving ICIs [24]. We visualised T cells (CD3-positive) and CXCL9-positive macrophages along with beta cells (insulin-positive) and alpha cells (glucagon-positive) in the islets of a cancer patient who developed type 1 diabetes following anti-CTLA-4 antibody (ipilimumab) and anti-PD-1 antibody (nivolumab) combination therapy (Fig. 5). The pancreas specimen, obtained 2 years after the initial incidence of ketoacidosis, had already lost beta cells in the islets (Fig. 5b [insulin]). However, the T cells (Fig. 5b [CD3]) and CXCL9-positive macrophages (Fig. 5b [CXCL9]) were observed among alpha cells (Fig. 5b [glucagon]) inside the islets. Such immune cell infiltrations were rarely observed in the islets of a control patient (Fig. 5a).

## Discussion

The present study demonstrated that the systemic application of MSCs protected male NOD mice from PD-1/PD-L1 blockade-induced diabetes. Repeated injection of the neutralising antibody against mouse PD-L1 caused a massive infiltration of immune cells in the islets and decreased the beta cell area and insulin content in the pancreas. Systemic MSC injection partially protected the pancreas from beta cell loss and preserved insulin content.

T cell-positive area was substantially increased by PD-1/PD-L1 blockade in the islets of MSC(−) NOD mice, similar to that in human islets in anti-PD-L1 antibody-induced type 1 diabetes [24]. It was recently reported that CD8-positive T cells secrete IFN-γ in response to PD-1 blockade, which in turn activates infiltrated monocyte-derived macrophages to accelerate diabetes progression [26]. Our present study recapitulated the previously reported massive T cell and CXCL9-positive macrophage accumulation in the islets in response to PD-1 blockade despite the use of different protocols and antibodies [26]. Importantly, T cells and CXCL9-positive macrophages were also observed in the islets of a cancer patient who received ICIs and developed type 1 diabetes (Fig. 5). We used male NOD mice, which are known not to develop diabetes, and found that T cells were already associated with the islets of control NOD mice. Although these islet-associated T cells in control mice were mainly located around the beta cell region, PD-1/PD-L1 blockade substantially increased T cell-positive area and, importantly, changed their surrounding localisation and led to a partial invasion of the deformed beta cell region. However, the most striking increase (over 40-fold) was observed in the area of CXCL9-positive macrophages in islets by PD-1/PD-L1 blockade in the MSC(−) group vs the control group (Fig. 3). These cells were localised mainly inside the beta cell region in the islets, in contrast to the localisation of the macrophages stained with Mac-2 antigen, which mainly occurred outside of the beta cells (Fig. 3). Notably, MSC treatment not only decreased the areas of T cells and CXCL9-positive macrophages but also changed their localisation, in particular, preventing accumulation in the beta cell region of the islets, suggesting that these cells have immunomodulatory effects. The presence of such types of immune cells in an actual diabetes patient following ICI treatments, but rarely in a control patient in this study (Fig. 5), will increase the feasibility of MSC treatment for this untreated diabetes.

Recently, macrophage-expressing C-X-C motif chemokine receptor 3 (CXCR3) ligands including CXCL9 were found to recruit CD8-positive T cells and be indispensable for anti-tumour efficacy of ICIs [29, 30], suggesting the importance of cytocidal macrophages working against both tumour cells and beta cells.

Macrophages are the cell type most actively targeted by exosomes [31], and MSCs protect organs from various diseases at least partly by secreting exosomes. We recently showed that exosome production by transplanted hMSCs is required for the treatment of heart failure in a mouse model of pressure overload-induced heart failure [20, 32]. The injected MSCs did not localise in the pancreas but abundantly in the lung tissue, suggesting an important role of secreted factors including cytokines and exosomes. Indeed, plasma exosome levels were strongly increased by MSC treatments. MSC-derived exosomes may suppress monocyte-derived macrophage recruitment and activation, thereby decreasing the incidence of diabetes induced by PD-1/PD-L1 blockade in male NOD mice. It will be intriguing in the future to test whether exosome production from injected hMSCs is required for the prevention of PD-1/PD-L1 blockade-induced diabetes in NOD mice.

ICI-associated type 1 diabetes is a less frequent but life-threatening endocrine side effect of cancer immunotherapy [2, 33]. In many cases, it develops in the initiation phase of ICI treatments and gradually progresses for months [8]. Importantly, exocrine pancreatic lipase and amylase frequently increase in the plasma before overt diabetes occurs, suggesting that there is a therapeutic window for the suppression of autoimmune attacks on islets [8]. Nonselective immunosuppressants such as steroids may counteract the cancer immunotherapy itself and have been reported to result in worse outcomes [34, 35]. Alternatively, adjuvant therapy can be introduced with the initiation of ICI treatments if such therapy has no or little effect on tumour immunity. The hMSCs did not completely prevent, but partially prevented, the occurrence of diabetes in our NOD mouse model. Because diabetes progressed within 2 weeks in this model, much faster than patients’ diabetes after ICI treatments, the clinical effectiveness of hMSCs may be greater. Furthermore, increasing adiponectin in patients will increase exosome production and secretion from MSCs and may enhance the therapeutic efficacy of MSCs if the effects rely on exosomes to some extent.

MSC therapy has increasingly been tested for several clinical applications. There have been no critical issues reported regarding cancer progression to date [36]. Several studies have shown the beneficial effects of MSC-based therapies for the treatment of different pathologies, including graft vs host disease, cardiac ischaemia and diabetes. While some studies indicate that MSCs may contribute to cancer pathogenesis, recent data have shown that MSCs instead have suppressive effects on cancer cells [36]. The results of a long-term retrospective study of patients with autoimmune disease suggested that MSC therapy is not necessarily related to the increased incidence of infections and cancers in patients with autoimmune disease, who are more likely to develop infectious diseases and cancers due to the use of immunosuppressants [37]. The ICIs are thought to be effective in up to 36.1% of cancer patients [38], while they brought diabetes in less than 1.4% of patients who received ICIs [3–7]. Therefore, beta cells seem to be much less frequent targets for this immune cytotoxic cascade than autologous tumours. We do not know how this cascade discriminates tumour cells and occasionally misunderstands autologous beta cells as targets. But if MSCs can improve this selectivity, then MSC therapy will bring new opportunities. The regulation of PD-L1 expression in beta cells may be an important clue.

The present study demonstrates the beneficial effect of MSC injections during ICI treatment and provides evidence that this adjuvant cell therapy could be evaluated clinically in the future. In summary, we showed that systemic MSC treatments effectively prevented PD-1/PD-L1 blockade-induced diabetes in male NOD mice.

## Supplementary information

ESM (PDF 3.41 MB)

## Data Availability

All datasets were deposited to 10.5061/dryad.xwdbrv1fh.

## Abbreviations

CTLA-4: Cytotoxic T-lymphocyte-associated protein 4
CXCL9: C-X-C motif chemokine ligand 9
hMSC: Human mesenchymal stem cell
HRP: Horseradish peroxidase
ICI: Immune checkpoint inhibitor
Mac-2: Macrophage-2
MFG-E8: Milk fat globule-EGF factor 8 protein
MSC: Mesenchymal stem cell
MSC(−): NOD mice which received anti-PD-L1 mAb without hMSCs
MSC(+): NOD mice which received anti-PD-L1 mAb and hMSCs
PBS(−): Dulbecco’s PBS without calcium and magnesium
PD-1: Programmed cell death protein 1
PD-L1: Programmed death-ligand 1
