# Supplementary material for: Human adipose-derived mesenchymal stem cells prevent type 1 diabetes induced by immune checkpoint blockade
Source: Diabetologia. 2022 May 5;65(7):1185–97. doi: 10.1007/s00125-022-05708-3 (PMC9174328; doi:10.1007/s00125-022-05708-3)
Supplement: Supplementary file 1 — (PDF 3.41 MB) [file 125_2022_5708_MOESM1_ESM.pdf]

ESM Fig. 1 Kawada-Horitani E et al.

(a)CD3/Insulin

Control1

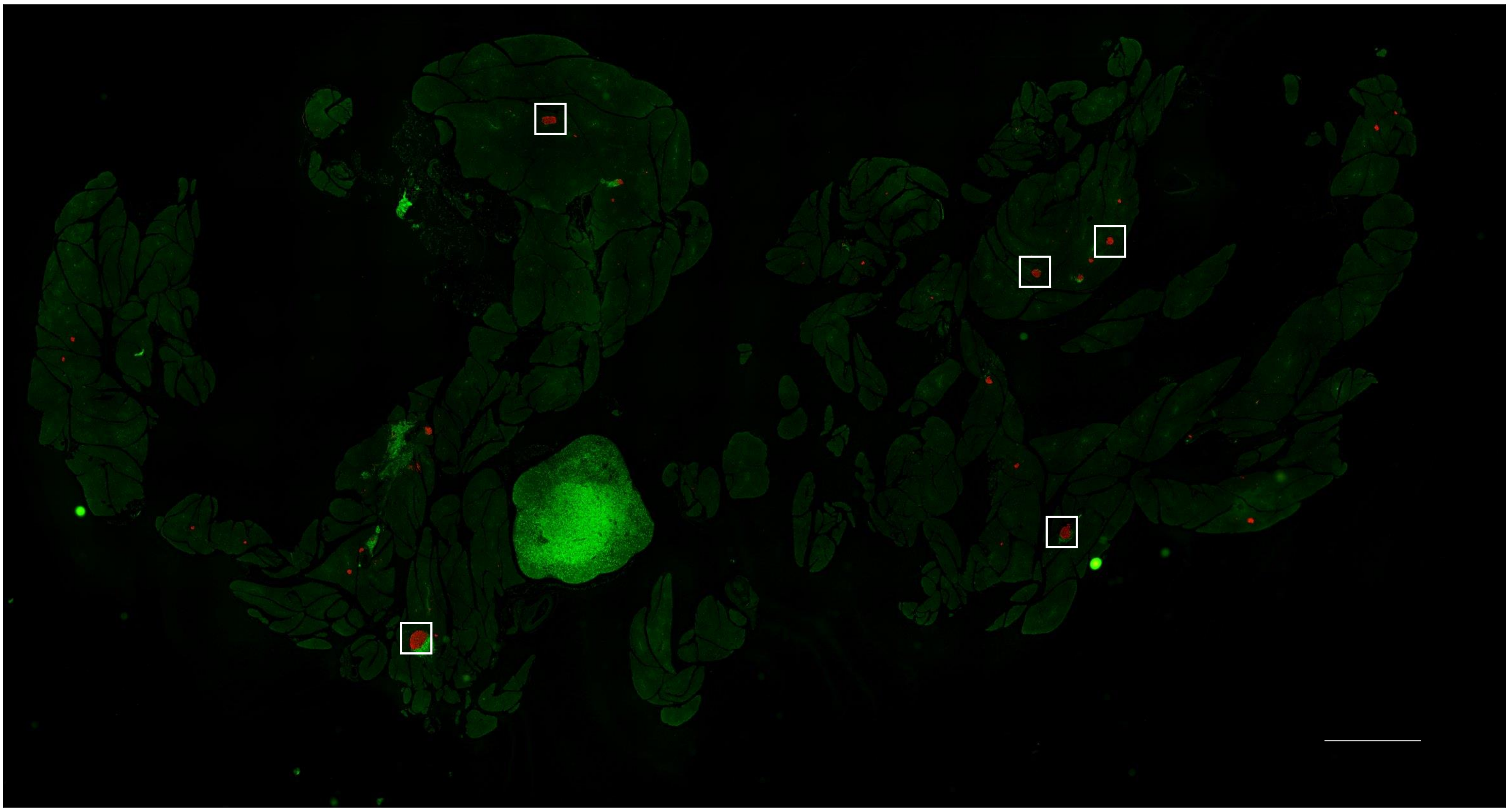

Control2

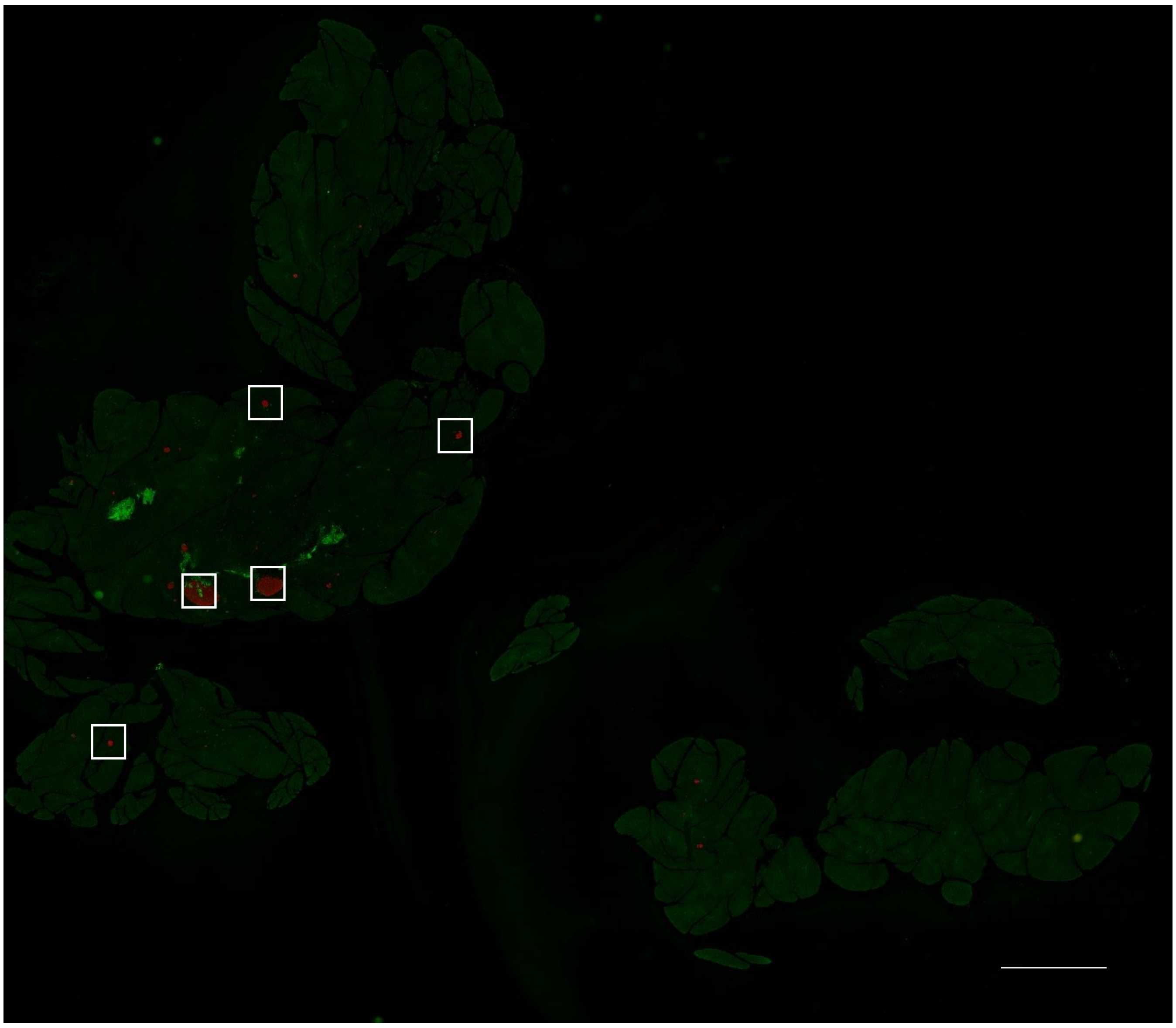

Control3

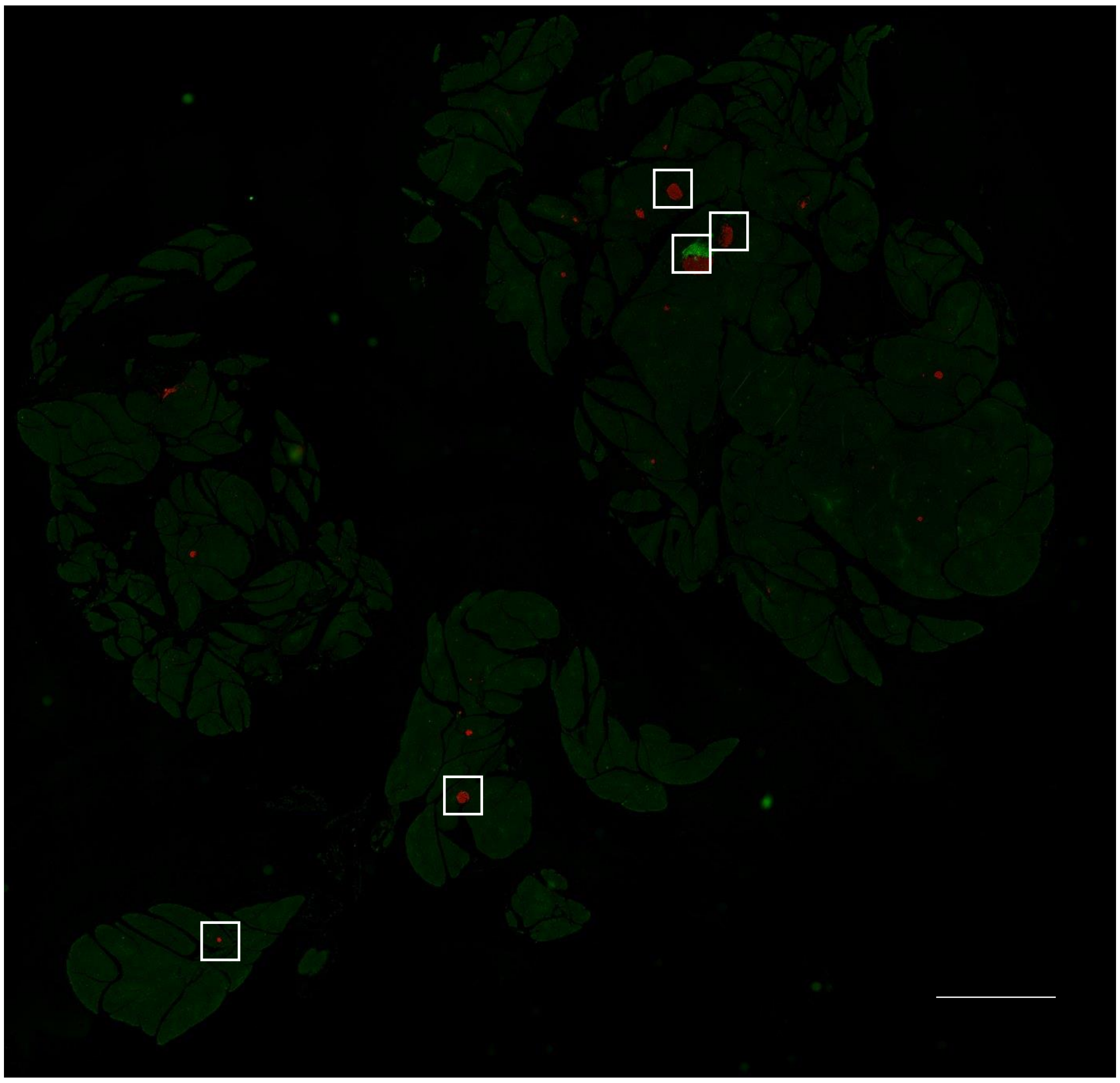

MSC(-) 1

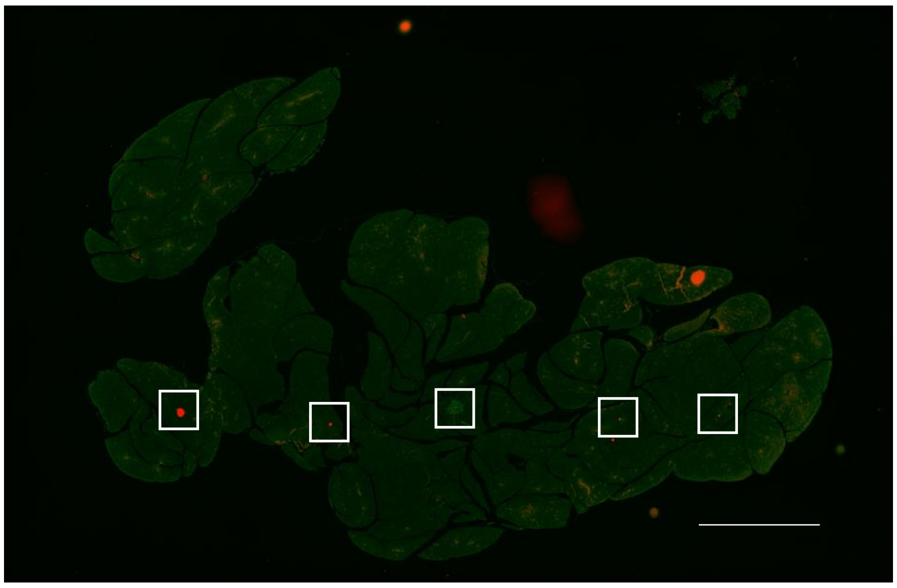

MSC(-) 2

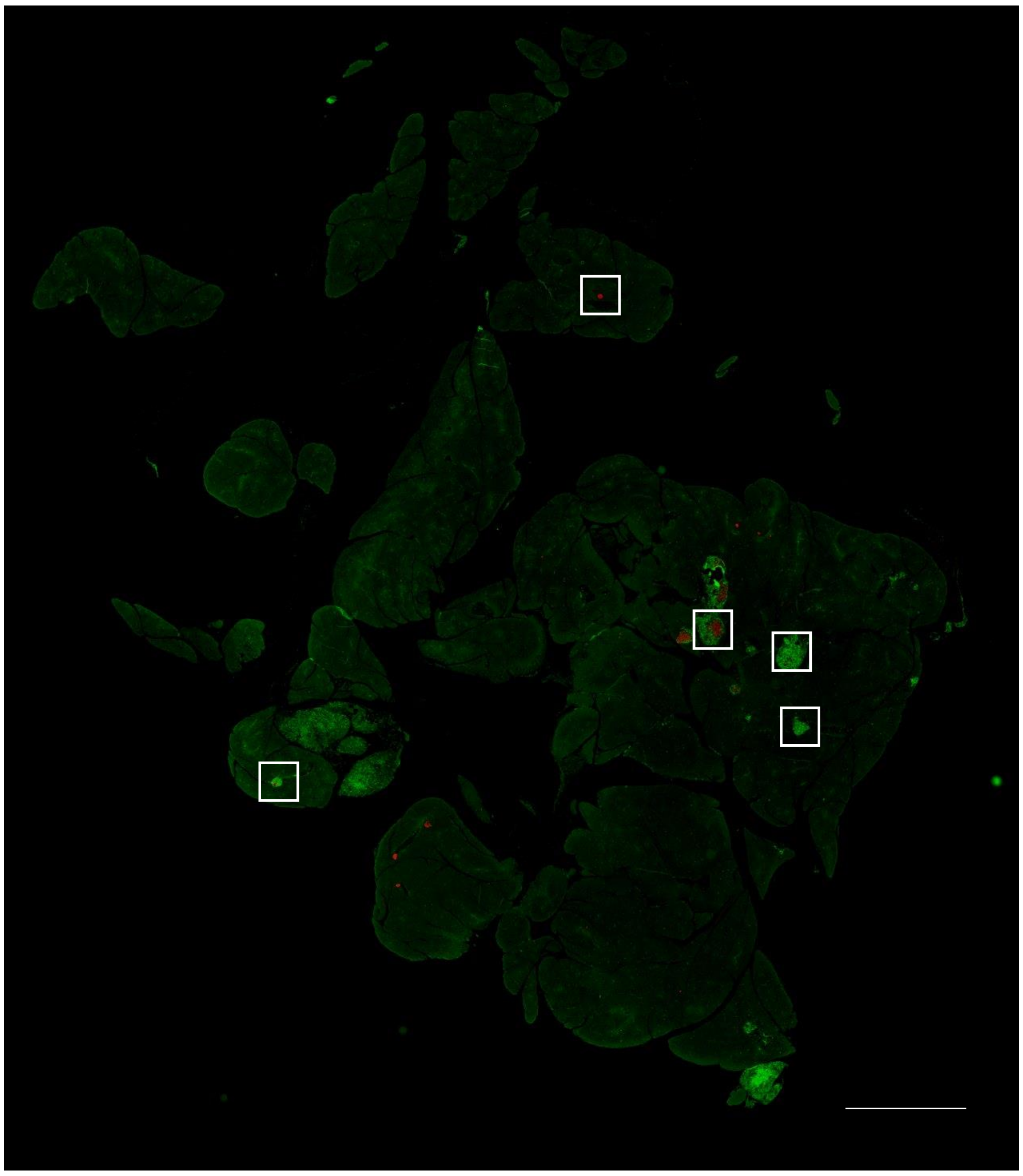

MSC(-) 3

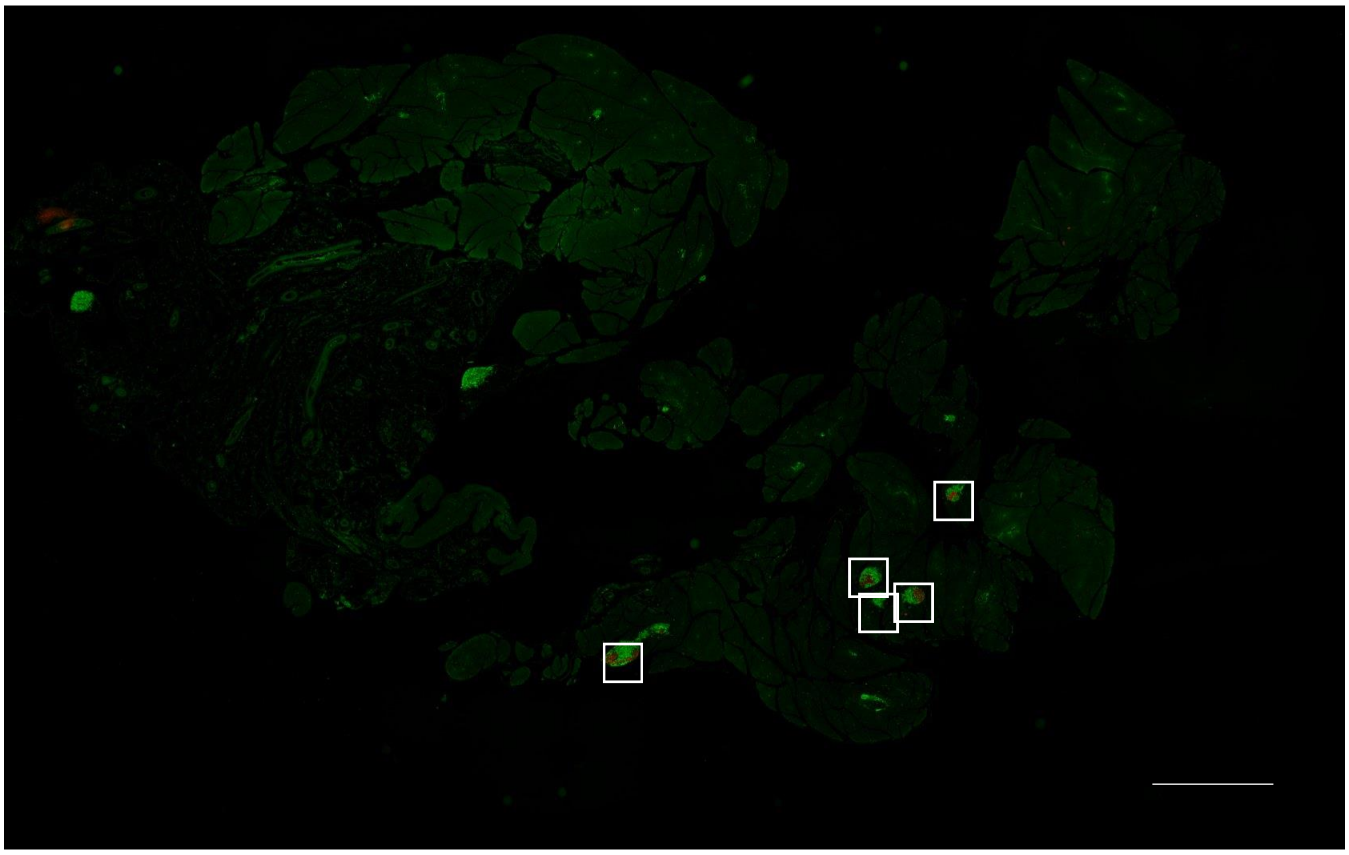

MSC(+) 1

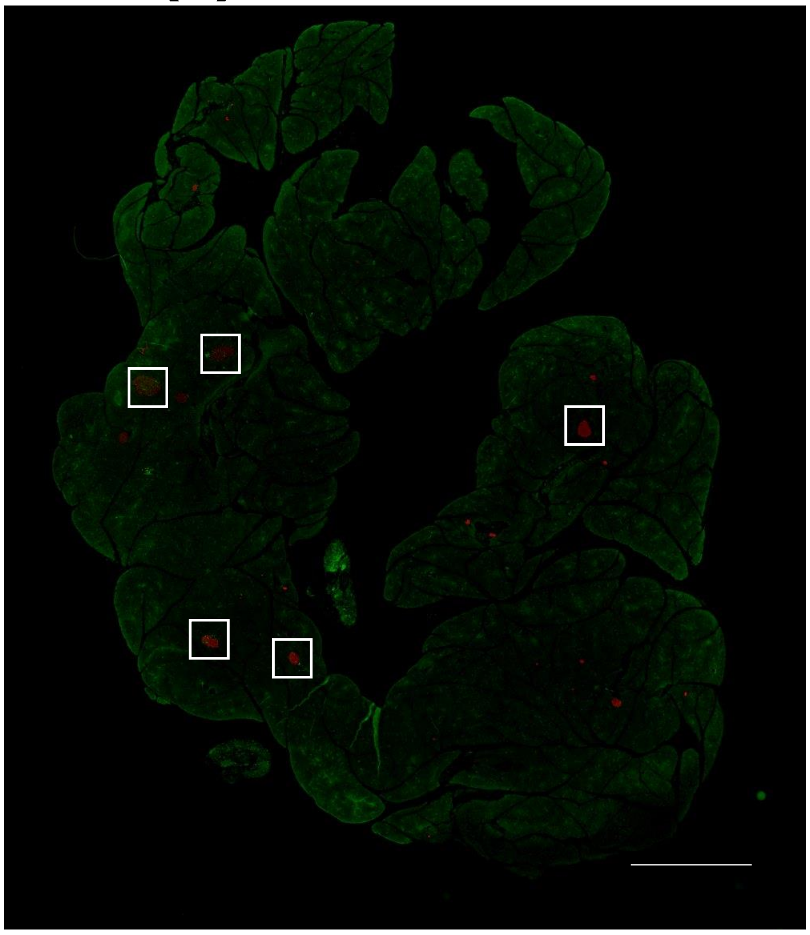

MSC(+) 2

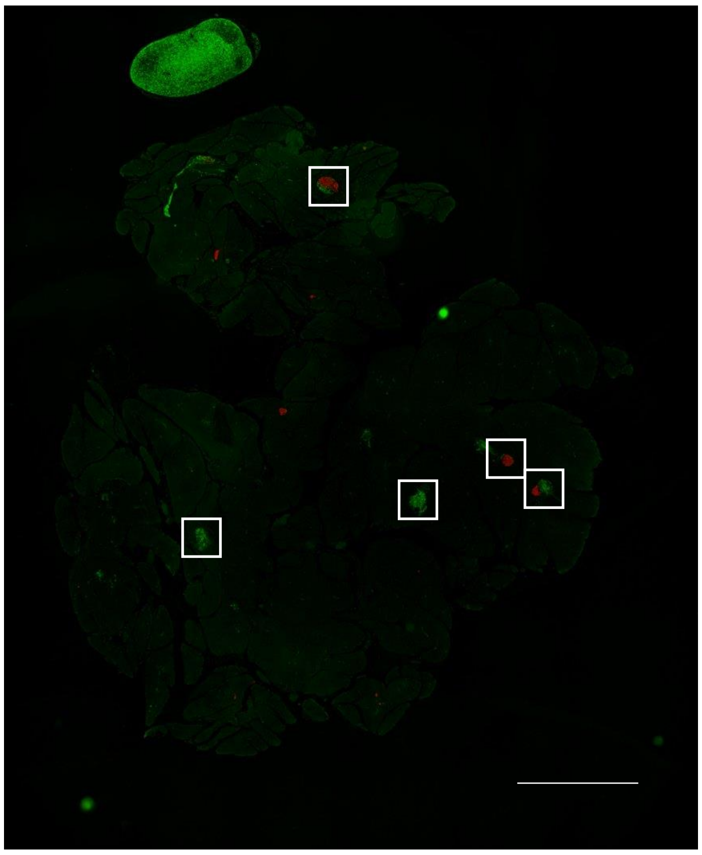

MSC(+) 3

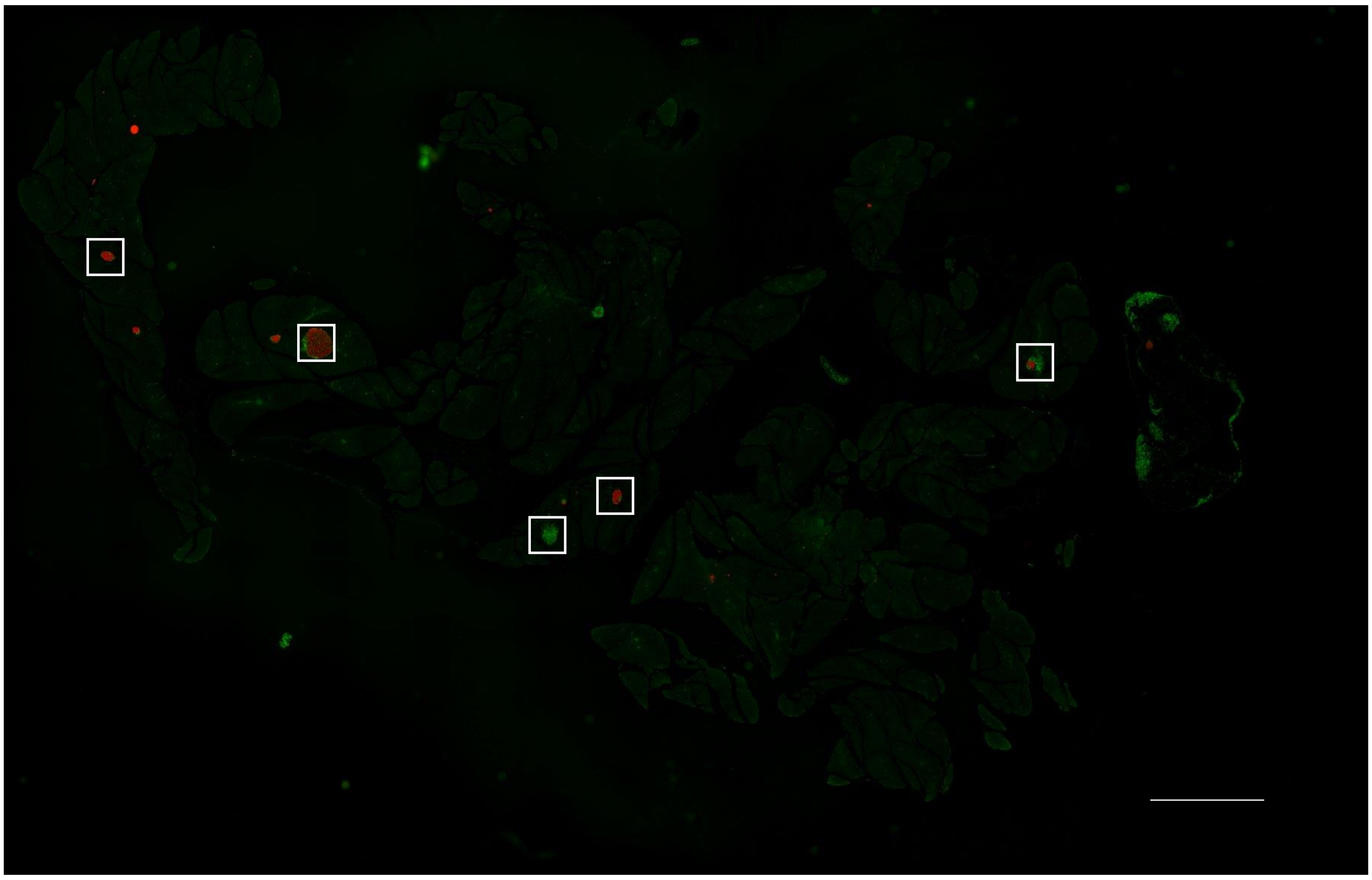

(b) Mac-2/Insulin

Control1

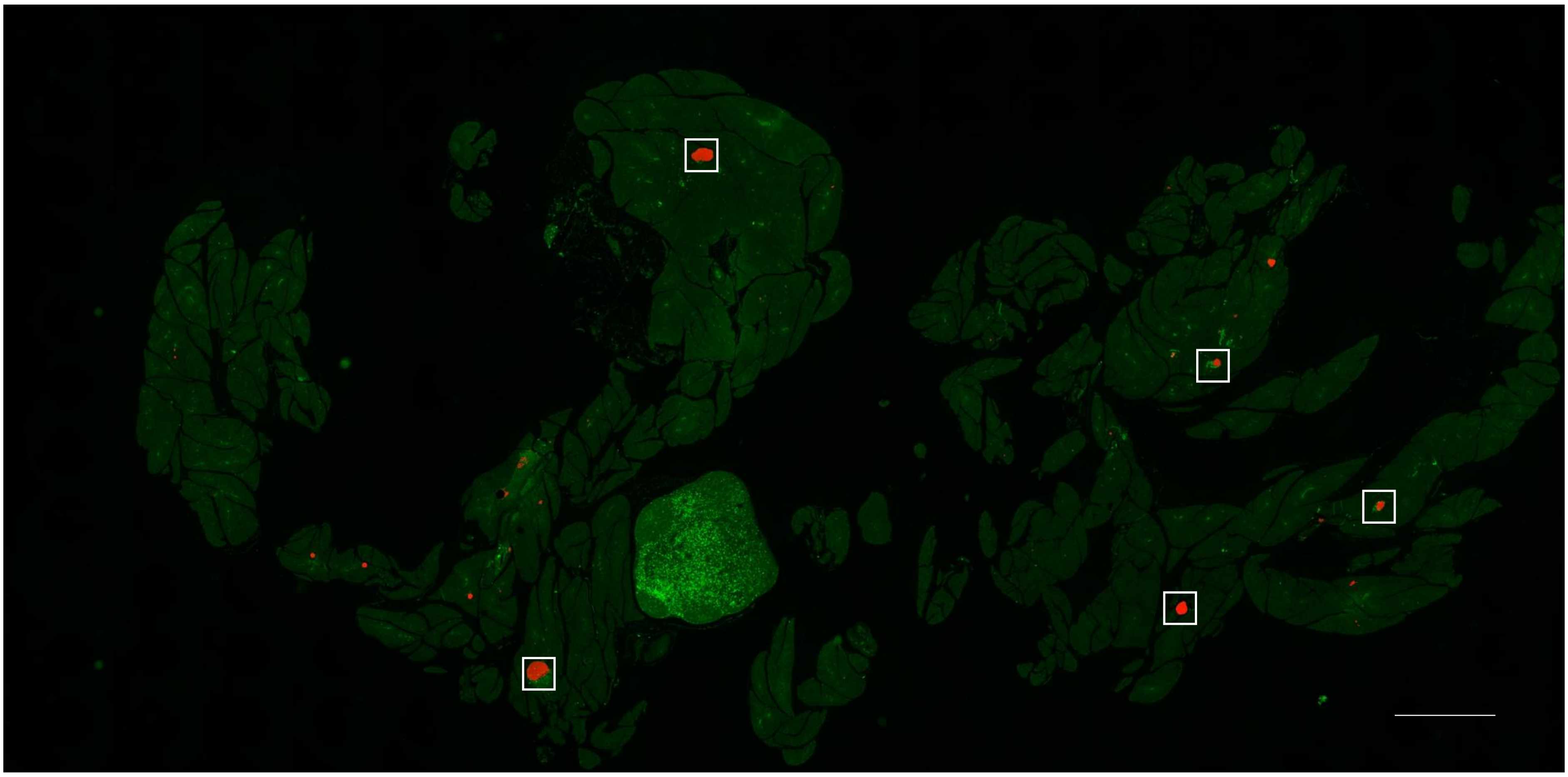

Control2

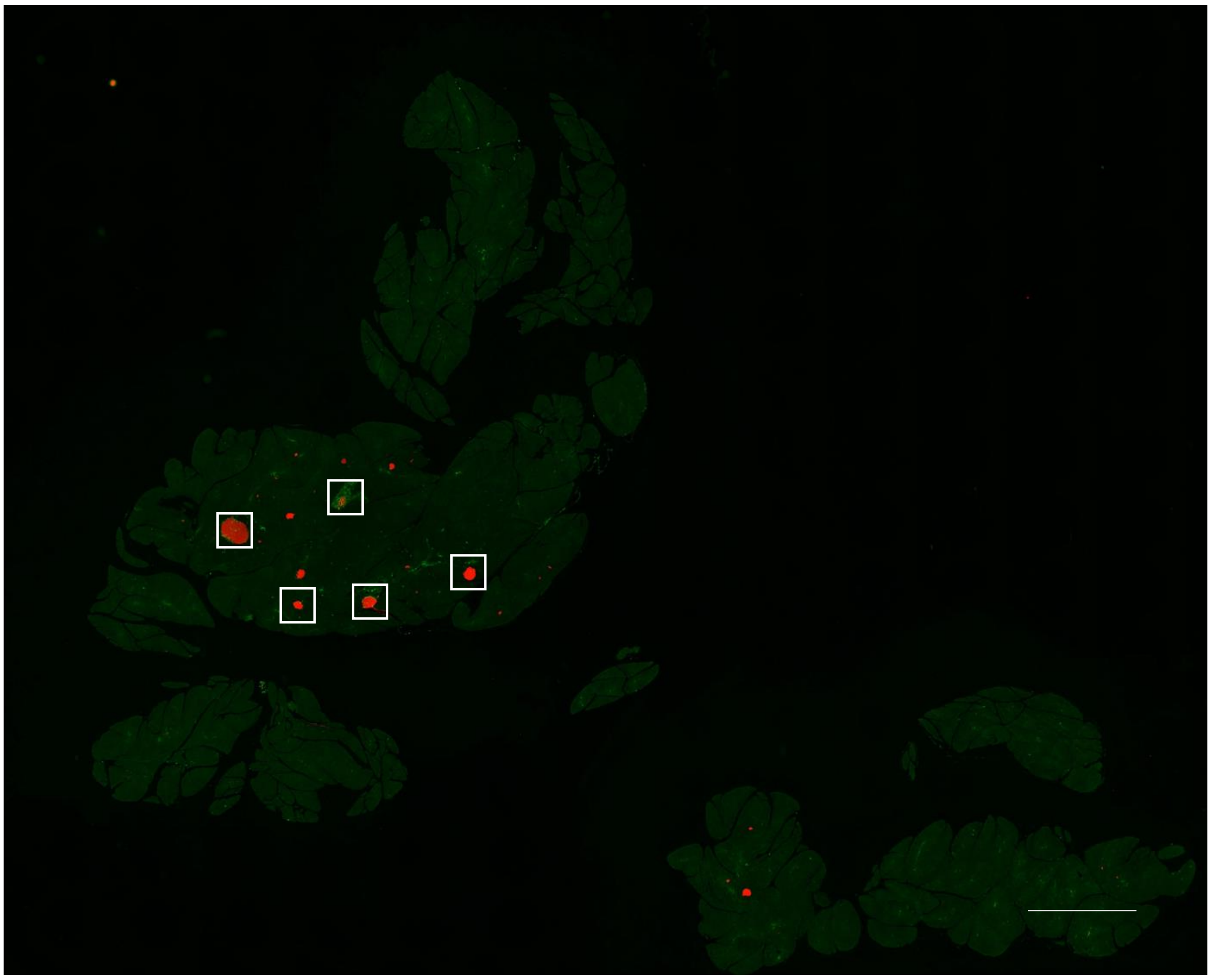

Control3

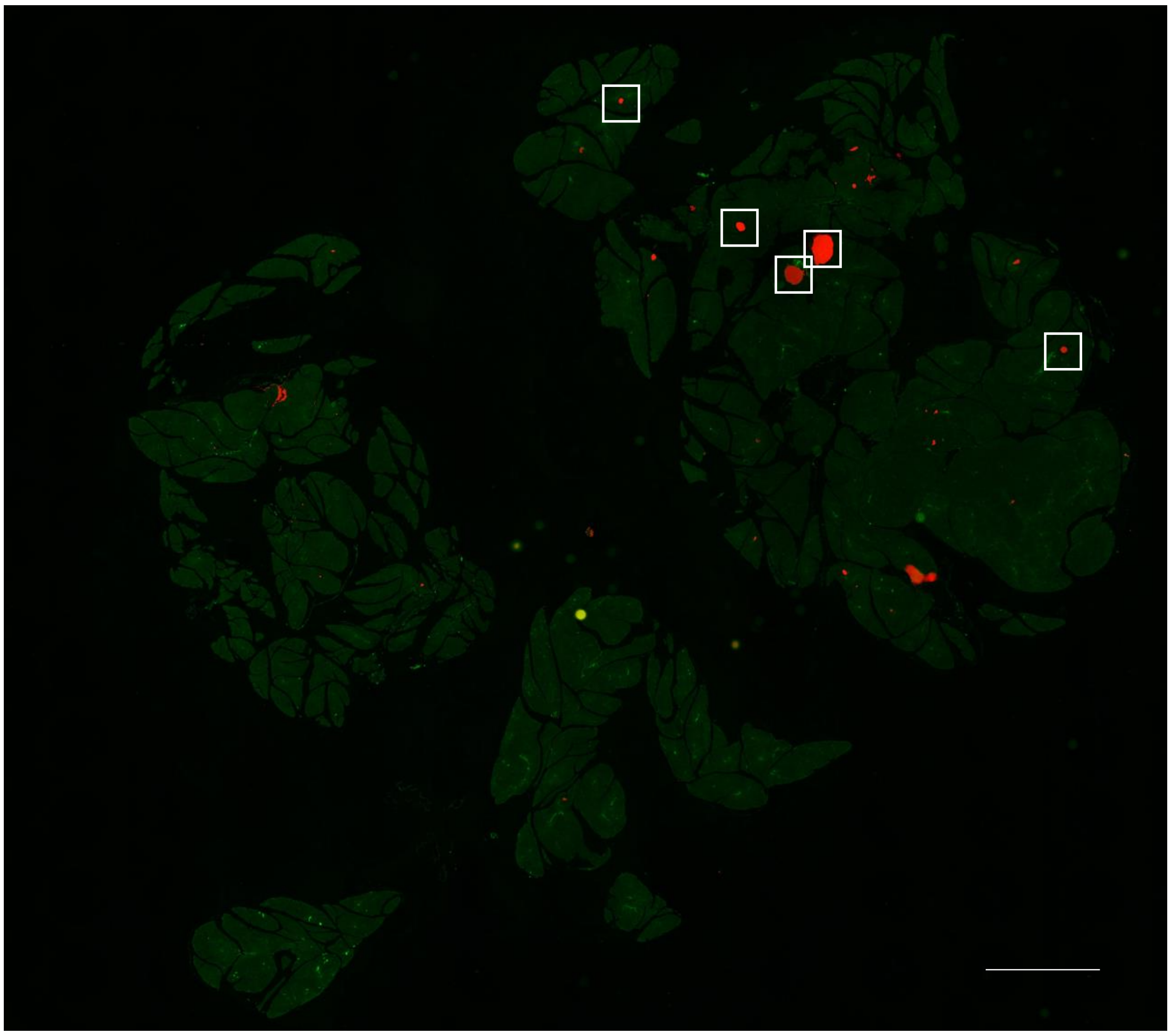

MSC(-) 1

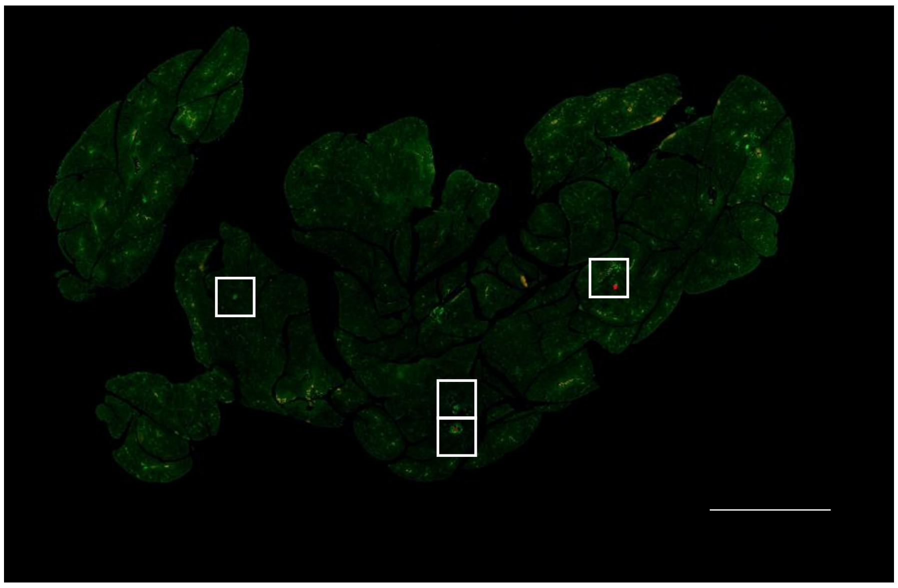

MSC(-) 2

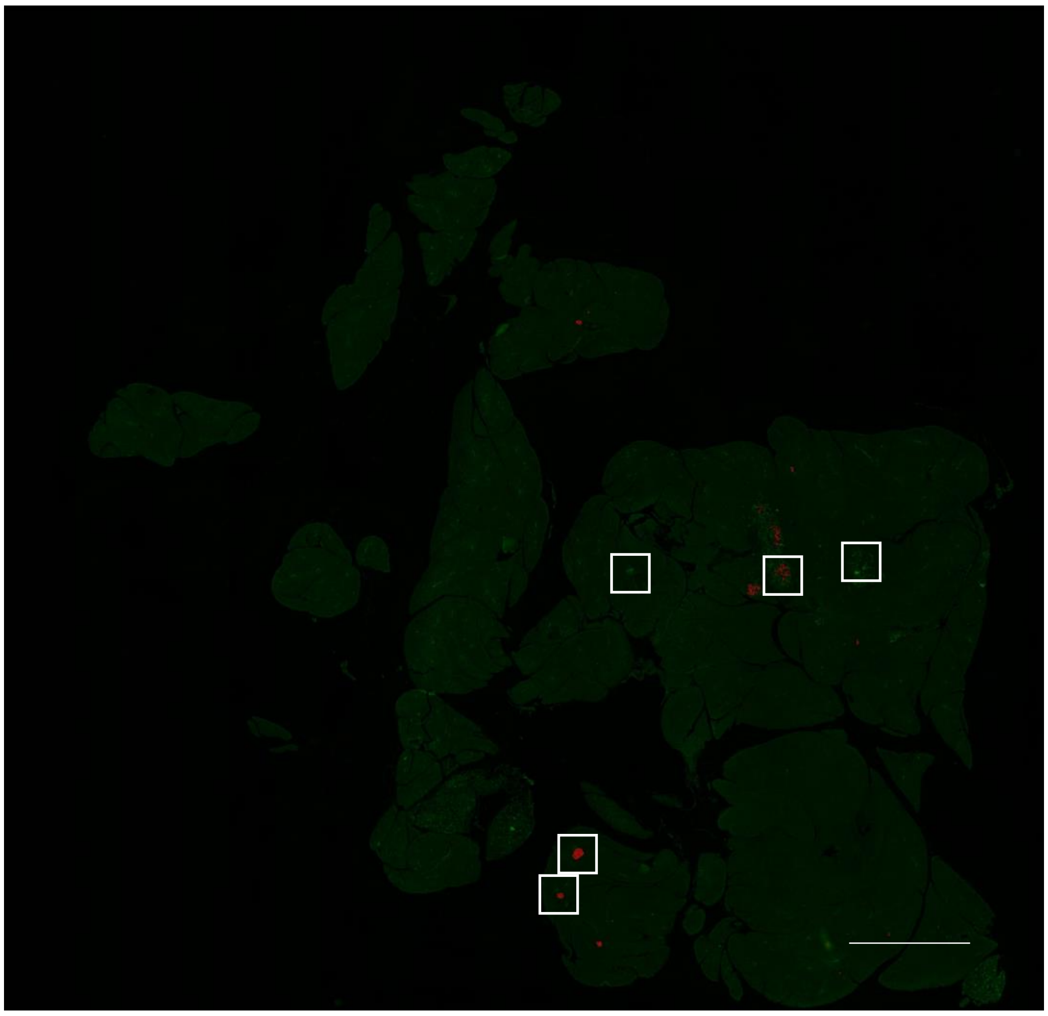

MSC(-) 3

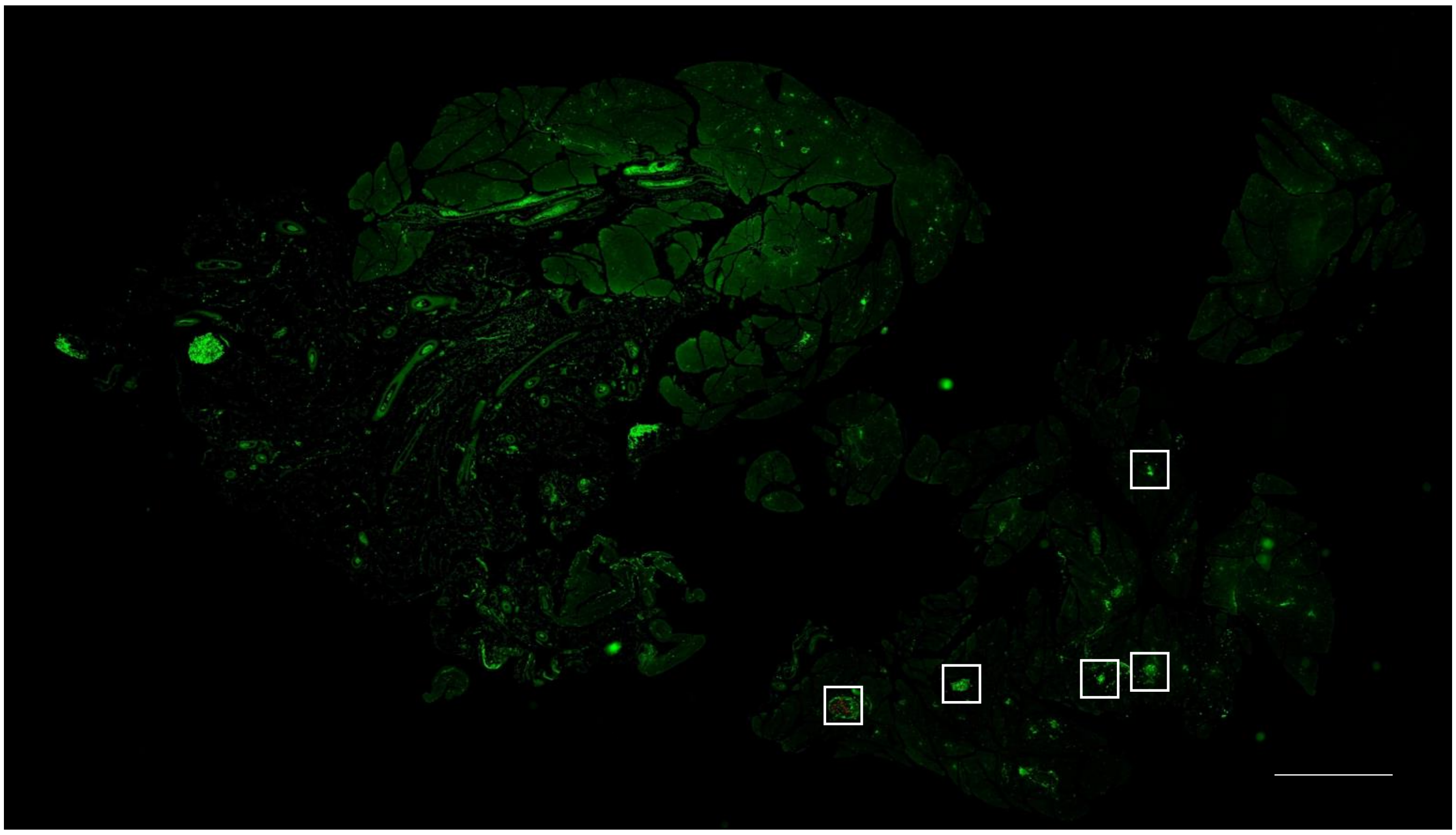

MSC(+) 1

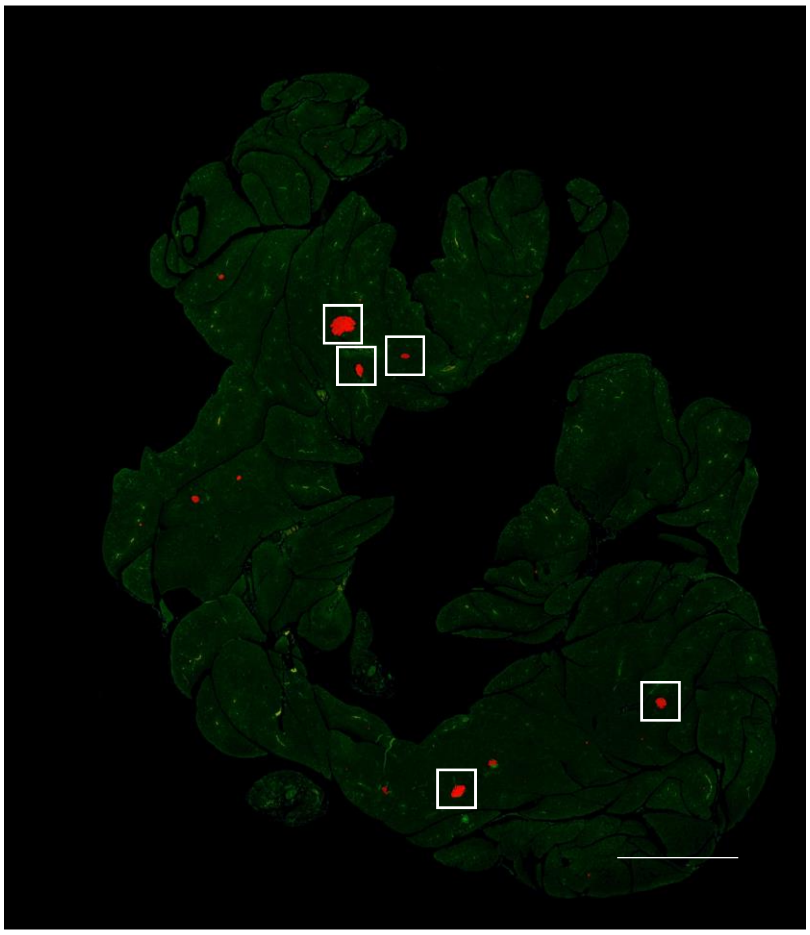

MSC(+) 2

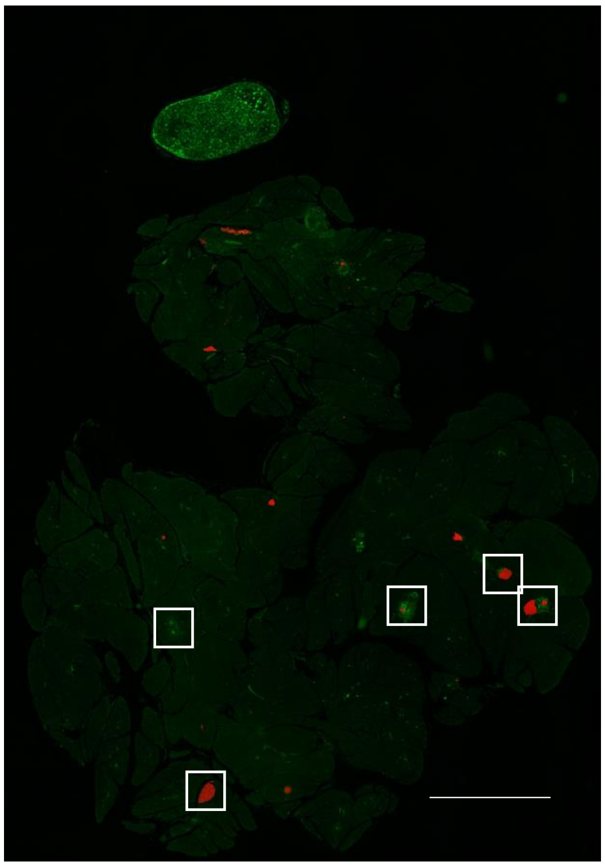

MSC(+) 3

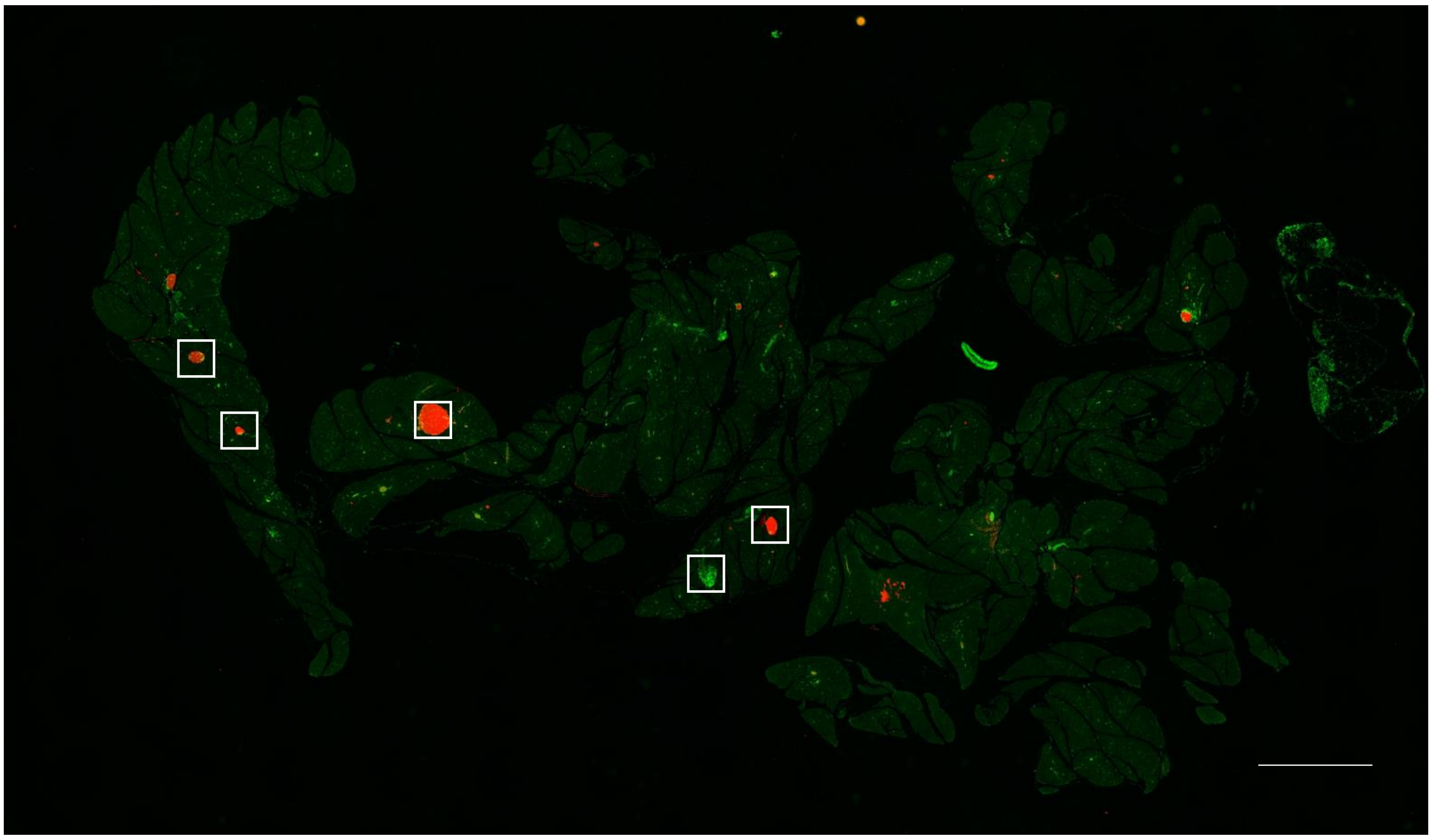

ESM Fig. 1 Kawada-Horitani E et al.

continue **(c) CXCL9/Insulin**

Control1

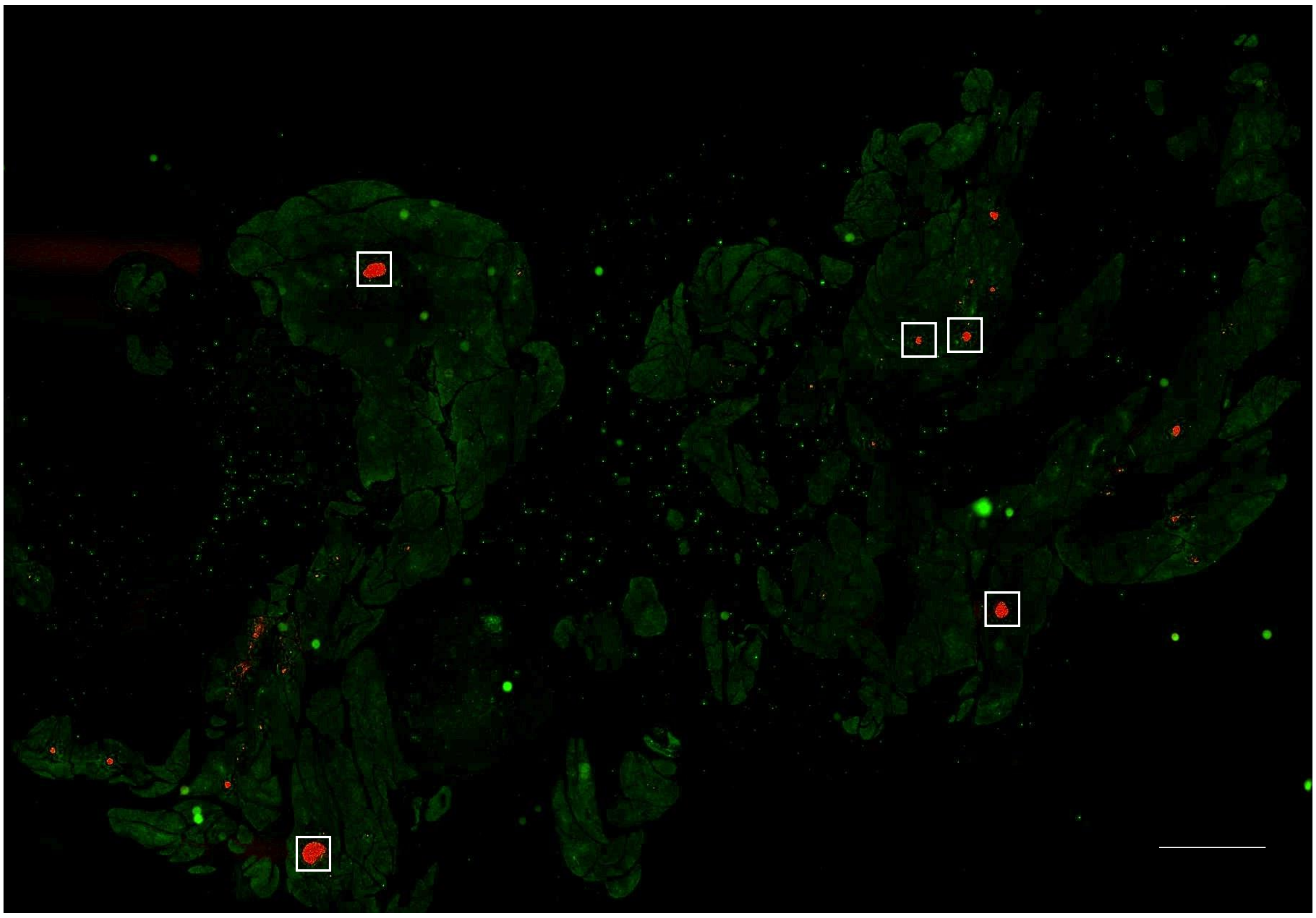

Control2

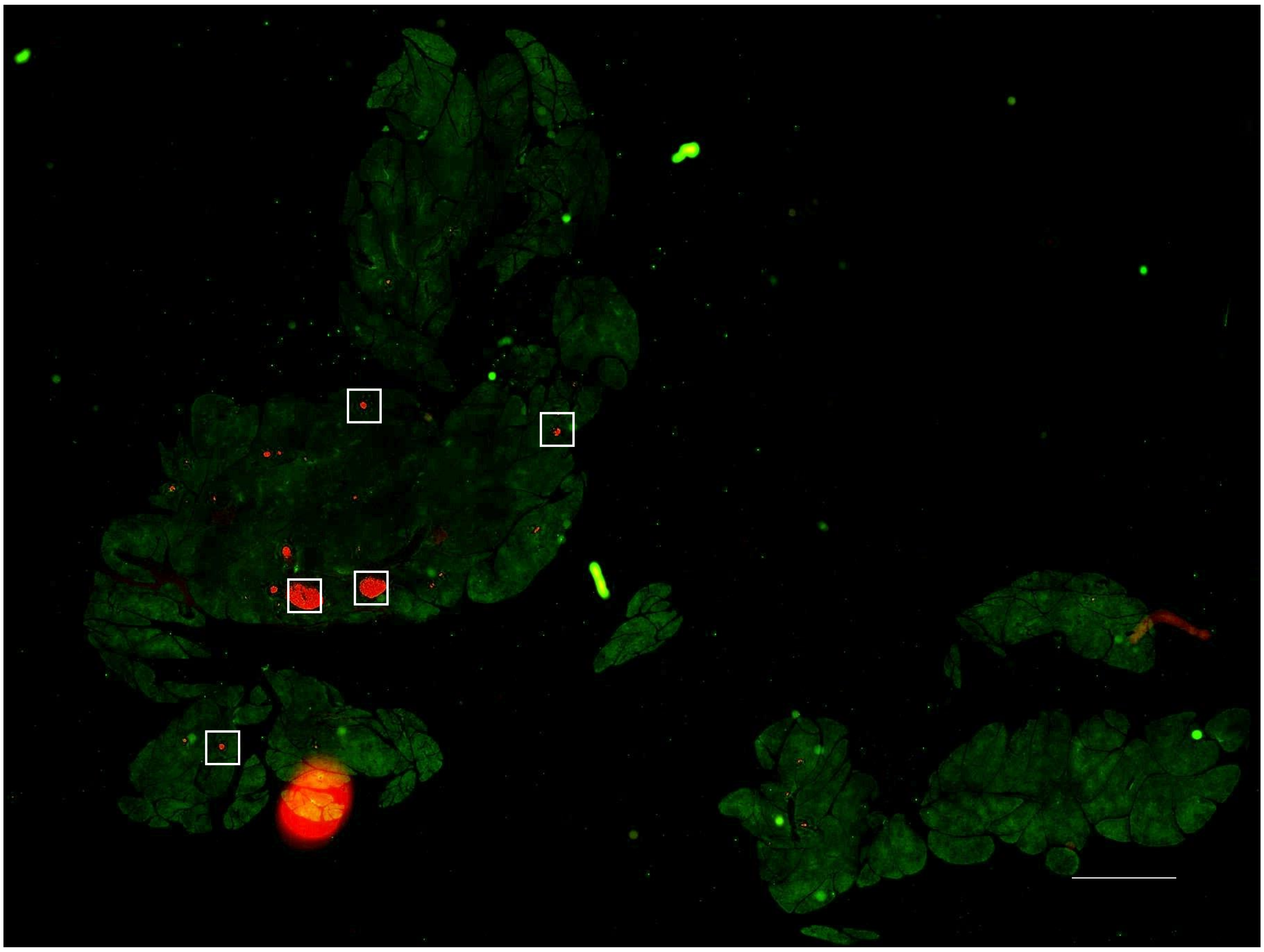

Control3

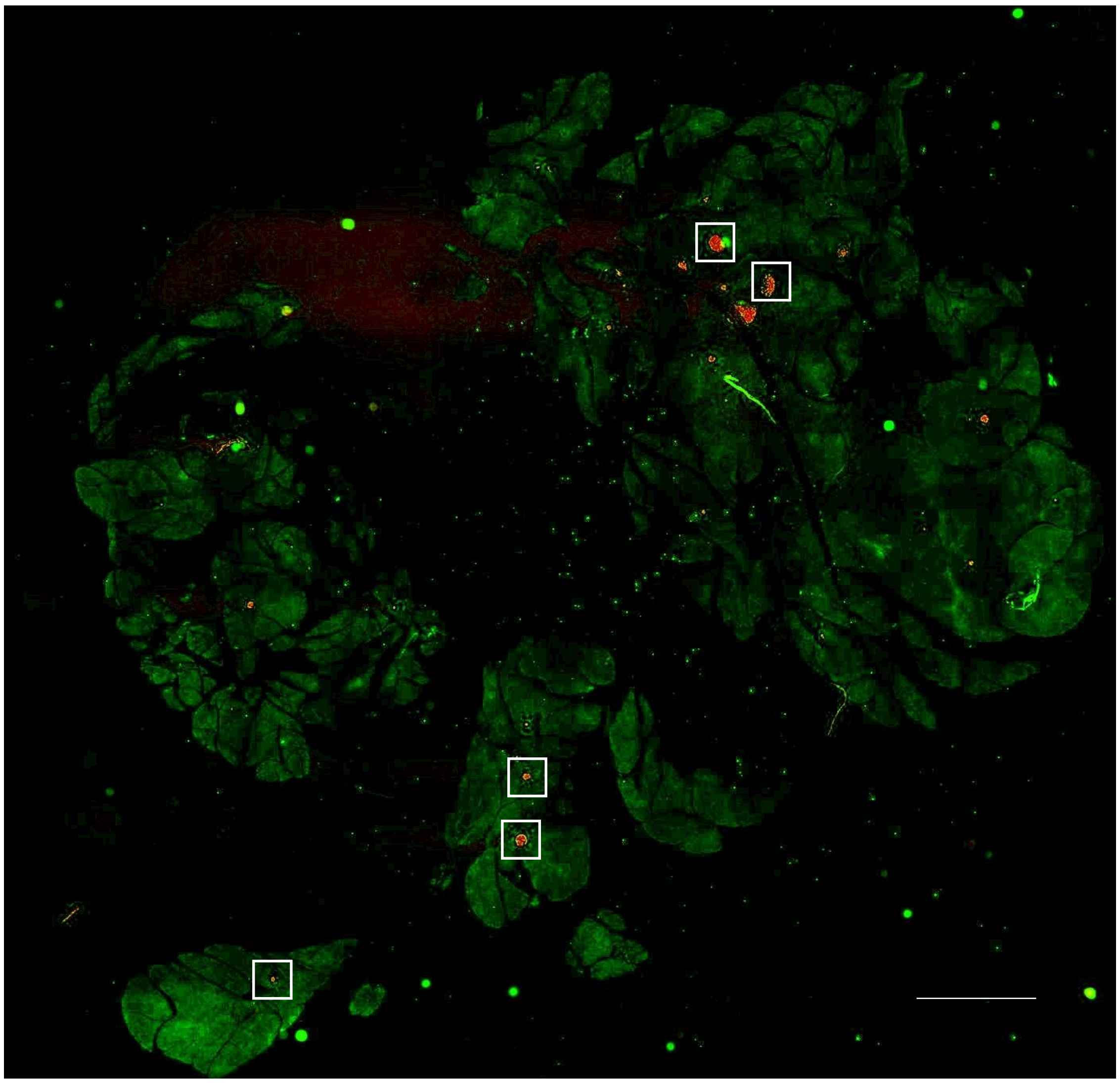

MSC(-) 1

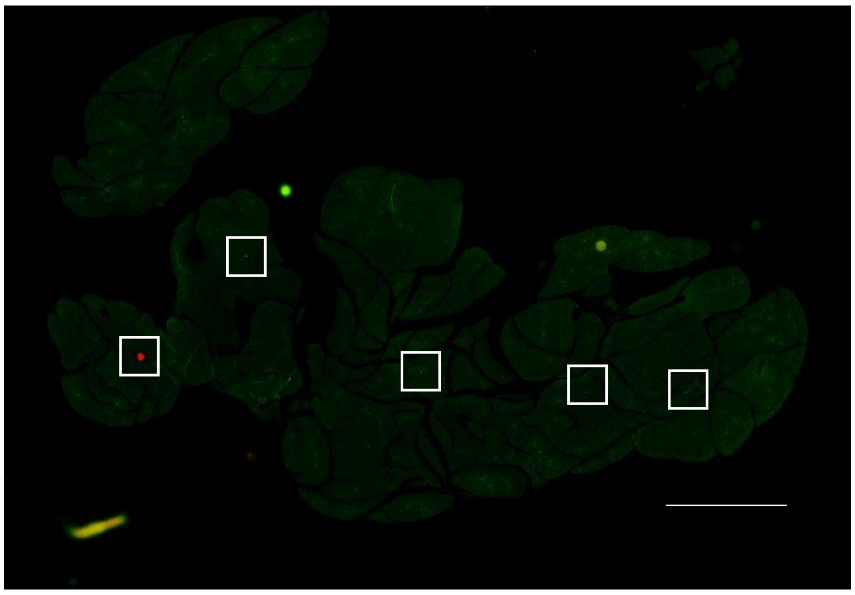

MSC(-) 2

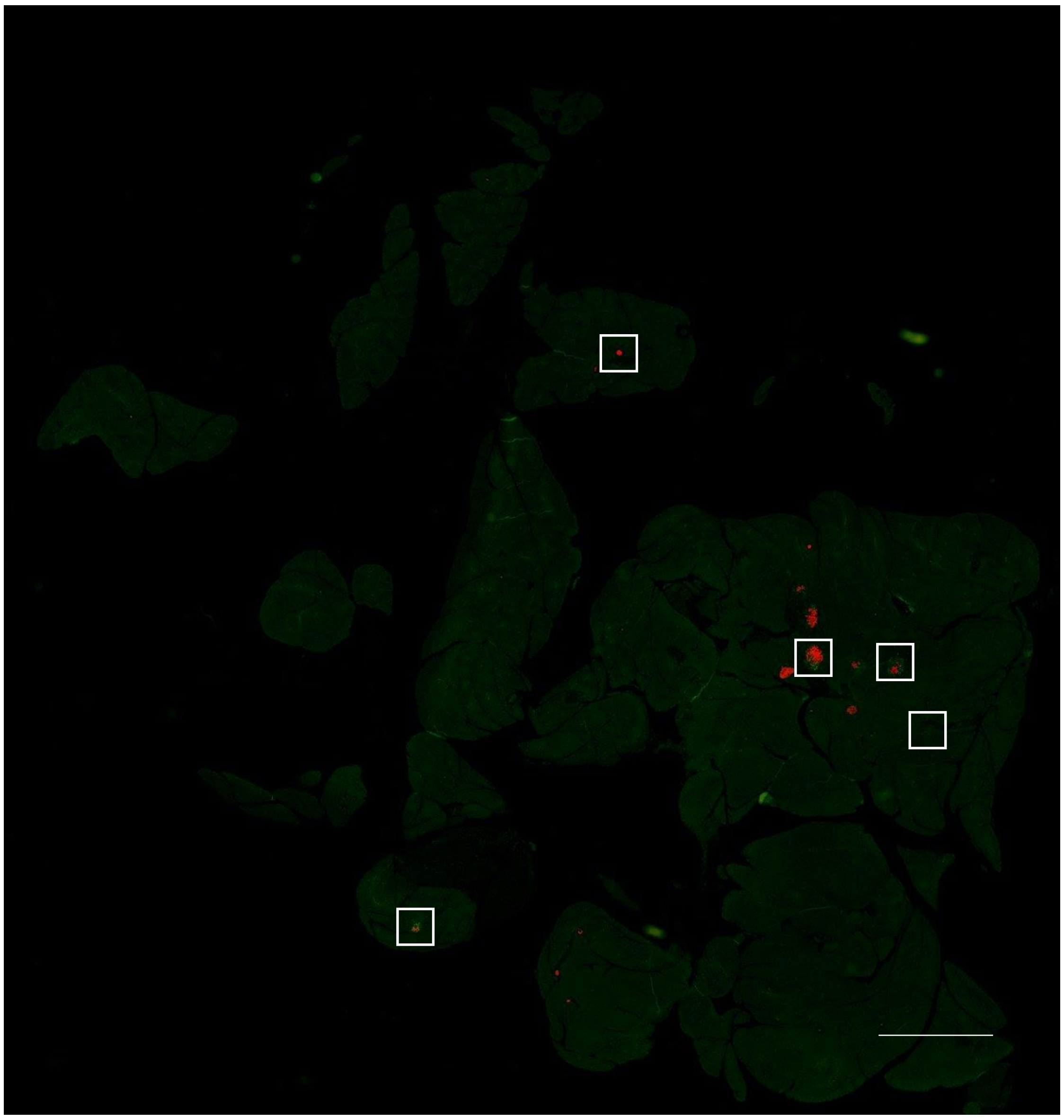

MSC(-) 3

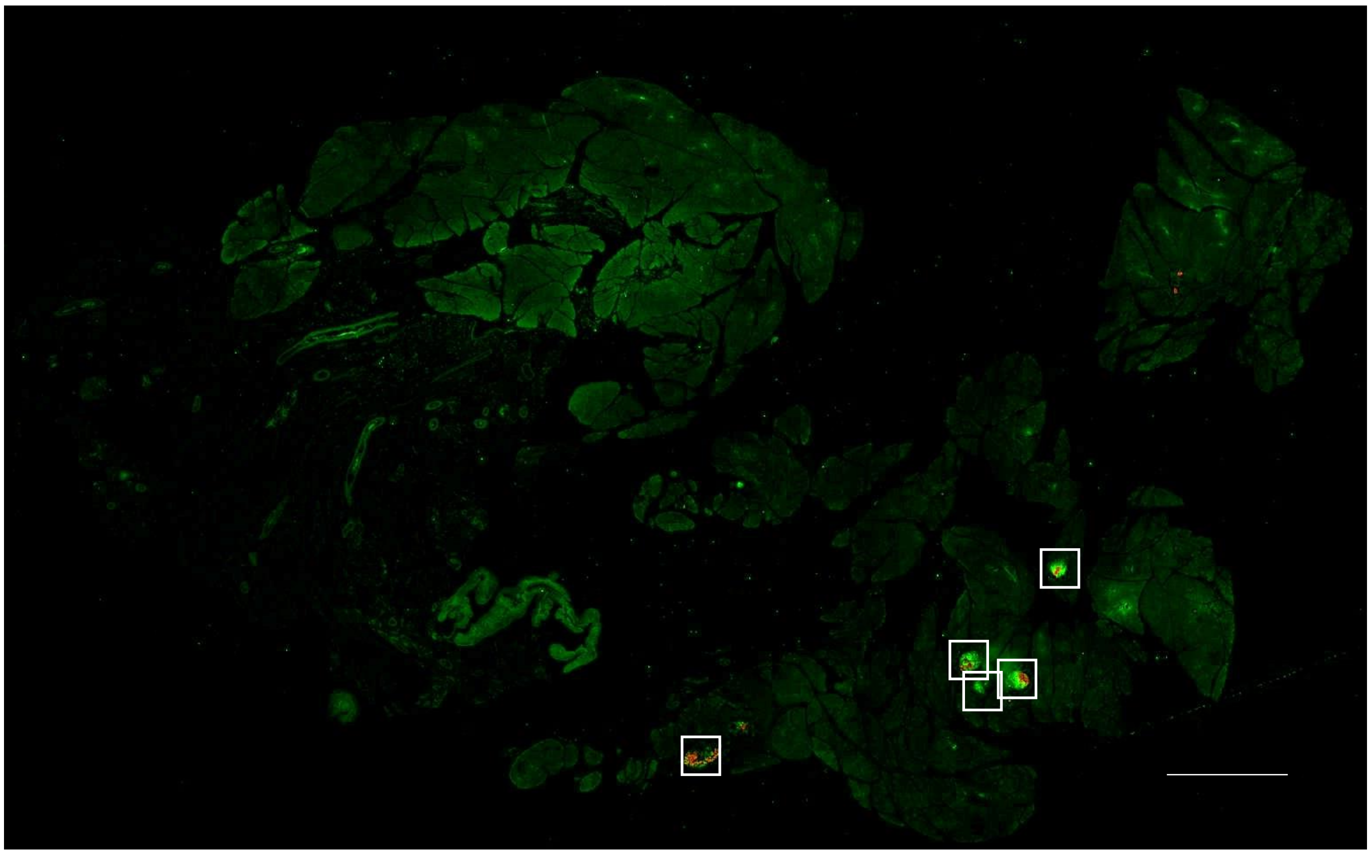

MSC(+) 1

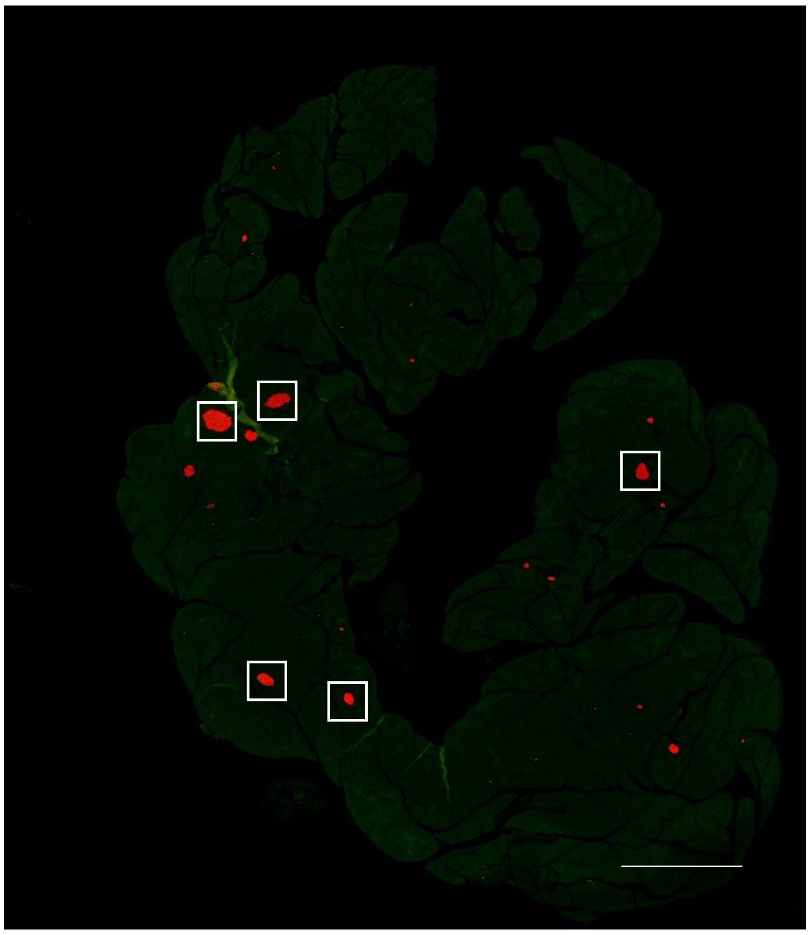

MSC(+) 2

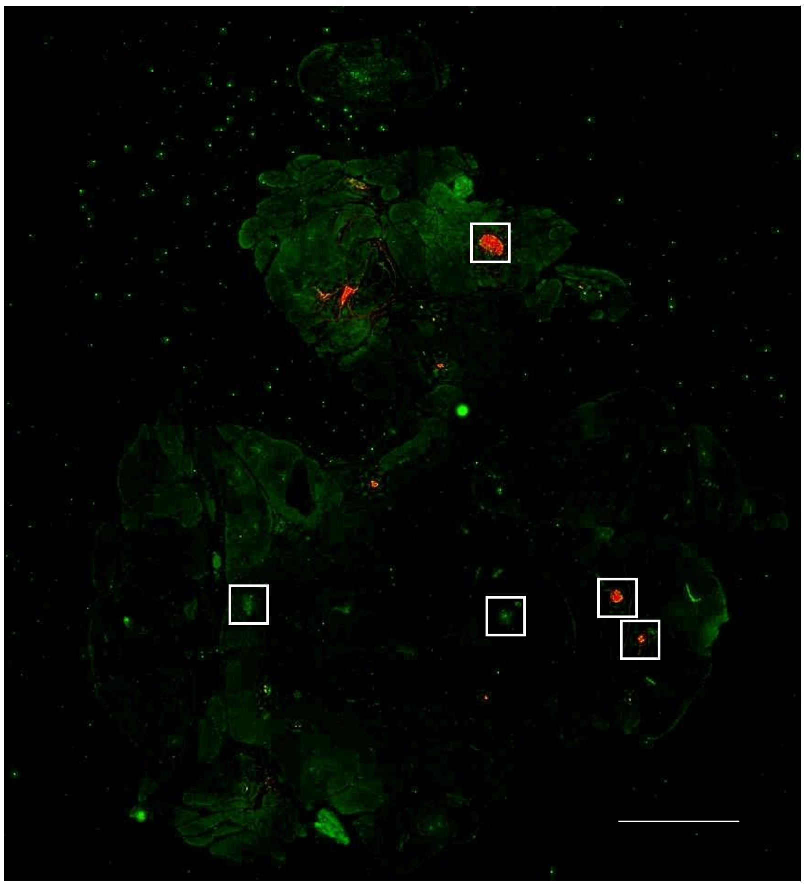

MSC(+) 3

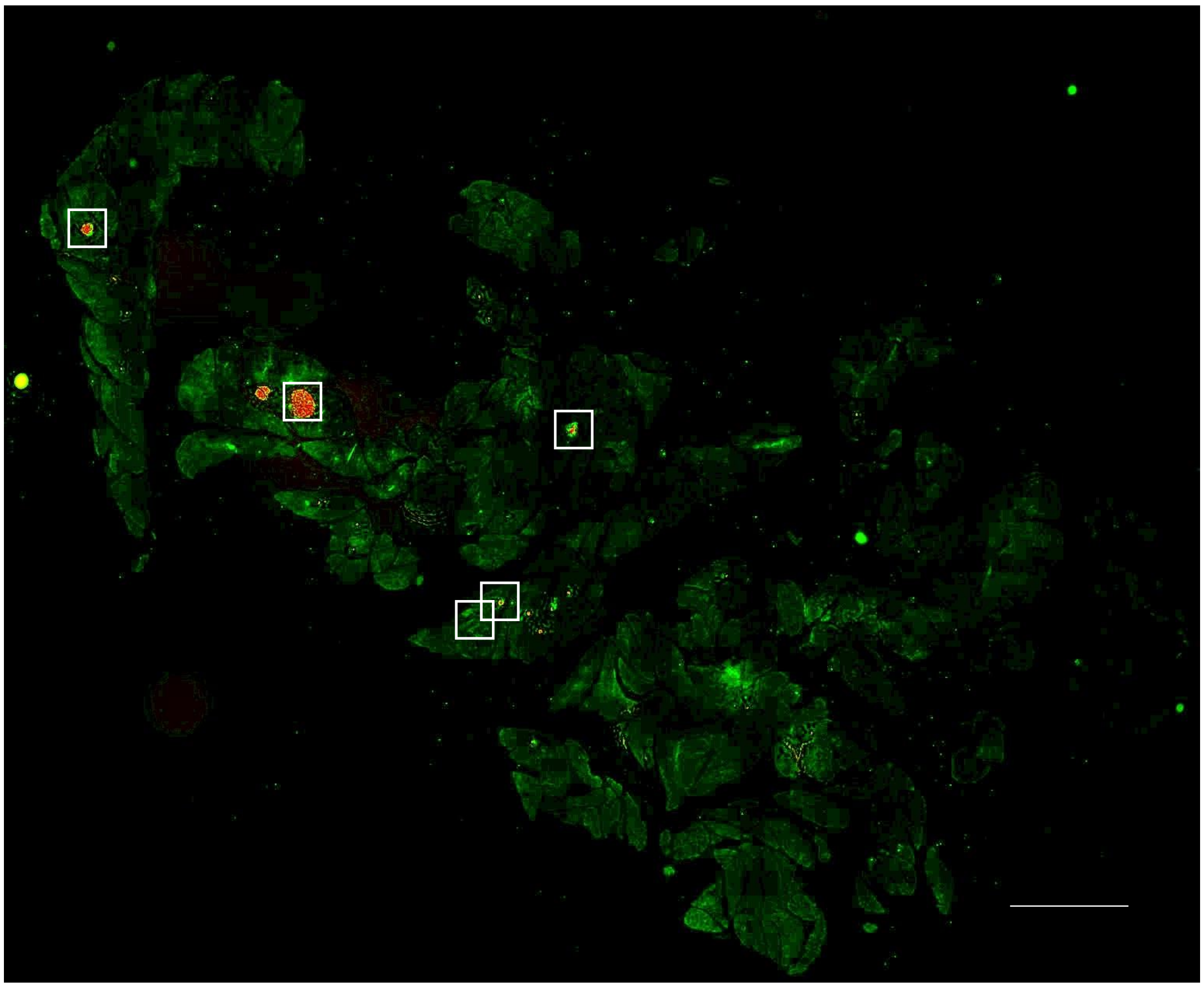

ESM Fig. 1. Macro images of islets used for quantitative evaluation of immunostaining.
Double immunofluorescence staining for (a) CD3 (green) and insulin (red), (b) Mac-2 (green) and insulin, and (c) CXCL9 (green) and insulin. Each location of the islets used for quantitative evaluation was indicated by a square. Scale bars, 1 mm. Control, NOD mice not receiving anti-PD-L1 mAb or hMSCs; MSC(-), NOD mice which received anti-PD-L1 mAb without hMSCs; MSC(+), NOD mice which received anti-PD-L1 mAb and hMSCs; Mac-2, Macrophage-2; CXCL9, C-X-C motif chemokine ligand 9

a control

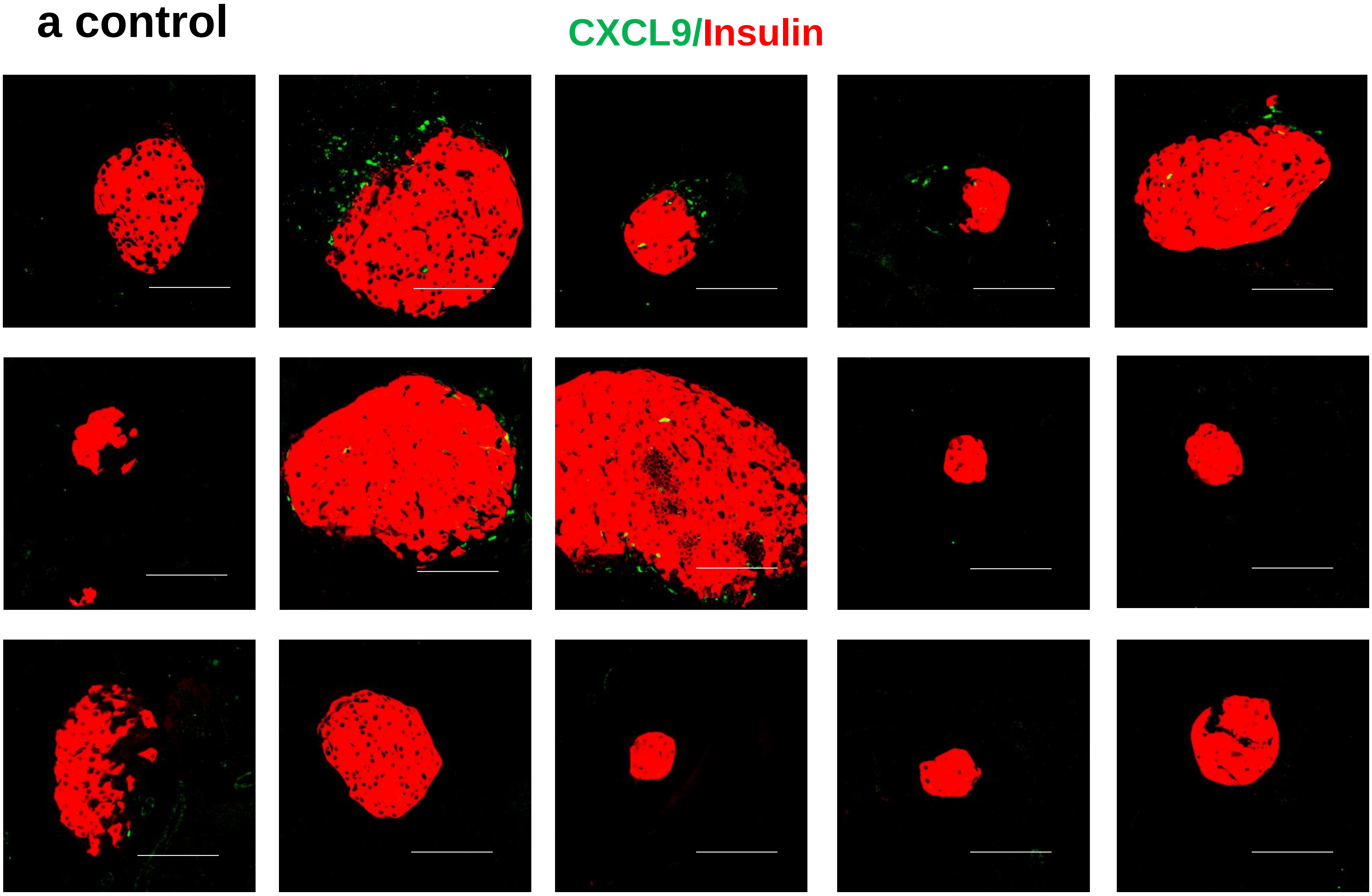

ESM Fig. 2 continue

Kawada-Horitani E et al.

b MSC(-)

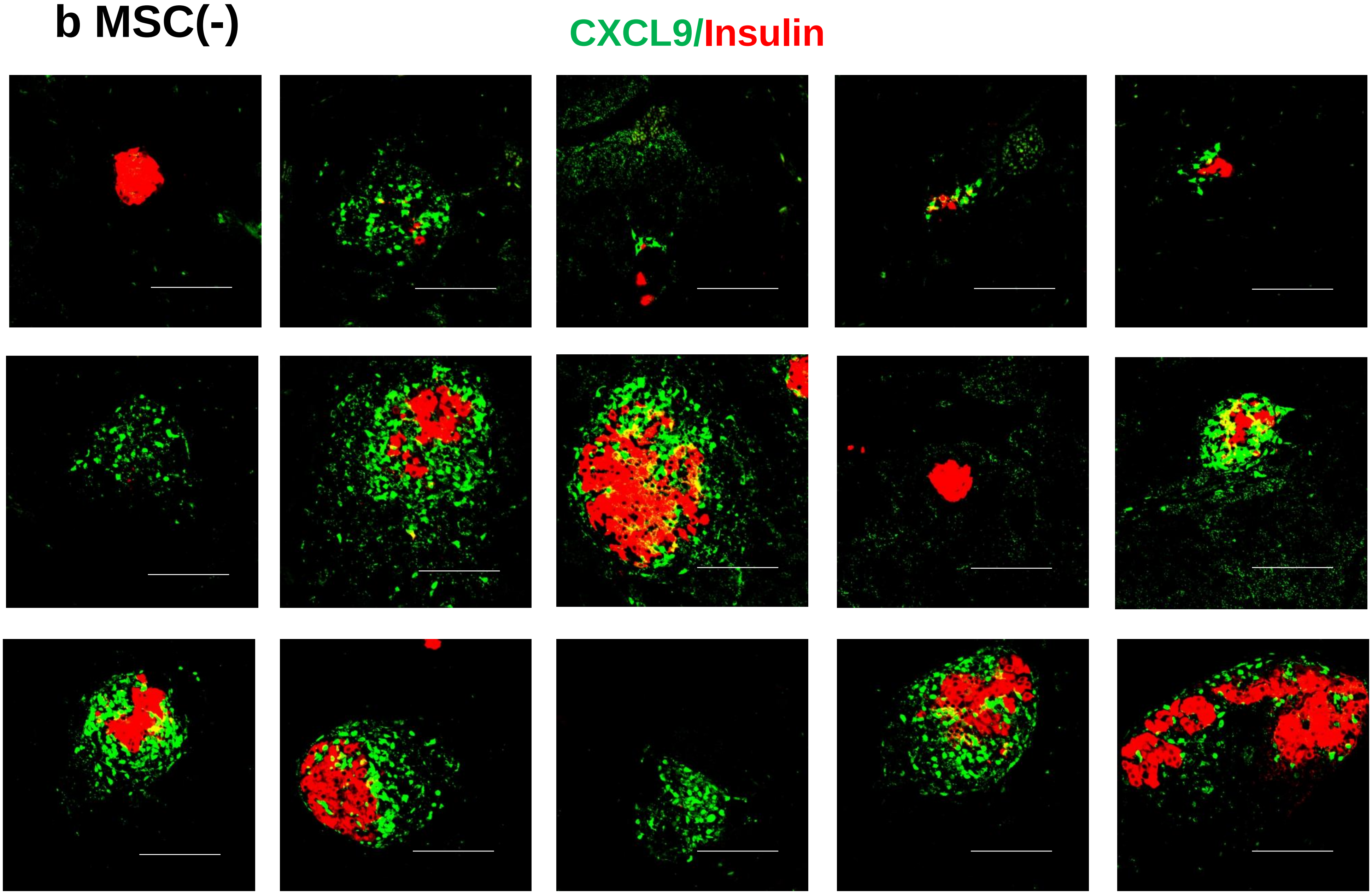

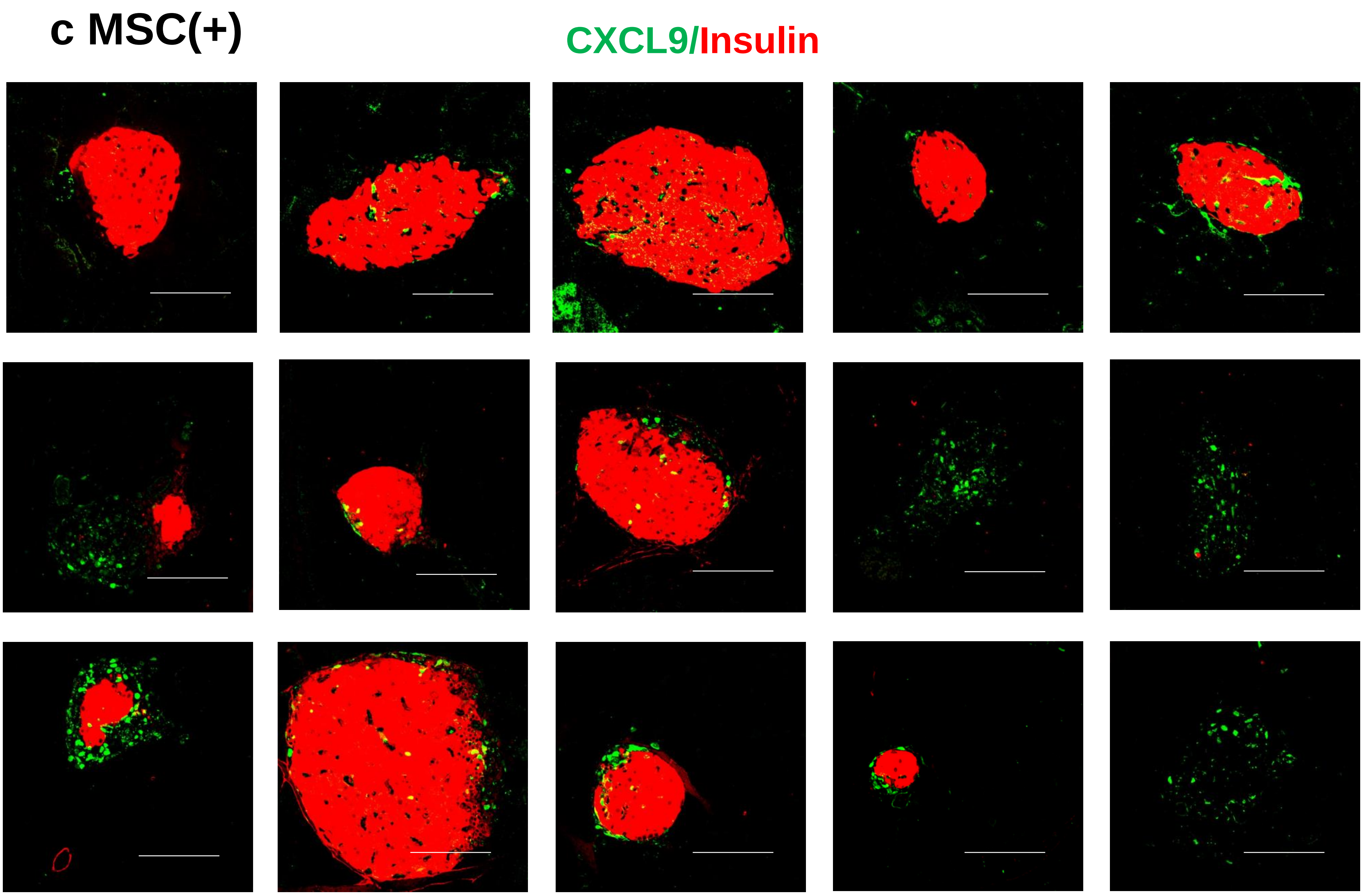

ESM Fig. 2. Enlarged images of the islets used for quantitative evaluation of immunostaining of CXCL9-positive macrophages.

Double immunofluorescence staining for CXCL9 (green) and insulin (red). (a) Islets from control group mice. (b) Islets from MSC(-) group mice. (c) Islets from MSC(+) group mice. Scale bars, 100 µm.

ESM Fig. 3

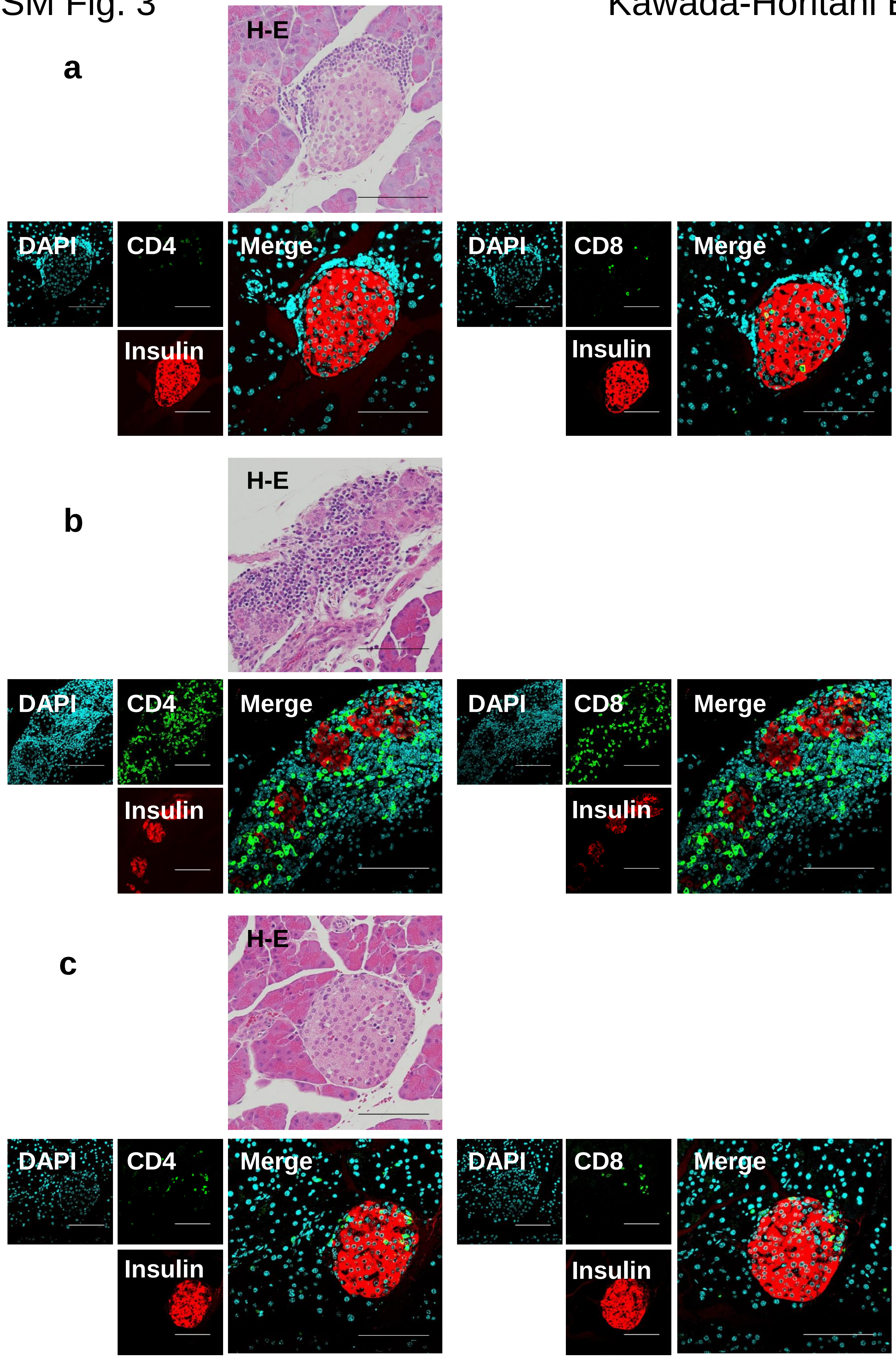
a
H-E
DAPI
CD4
Merge
Insulin
DAPI
CD8
Merge
Insulin
b
H-E
DAPI
CD4
Merge
Insulin
DAPI
CD8
Merge
Insulin
c
H-E
DAPI
CD4
Merge
Insulin
DAPI
CD8
Merge
Insulin

ESM Fig. 3 continue

Kawada-Horitani E et al.

d

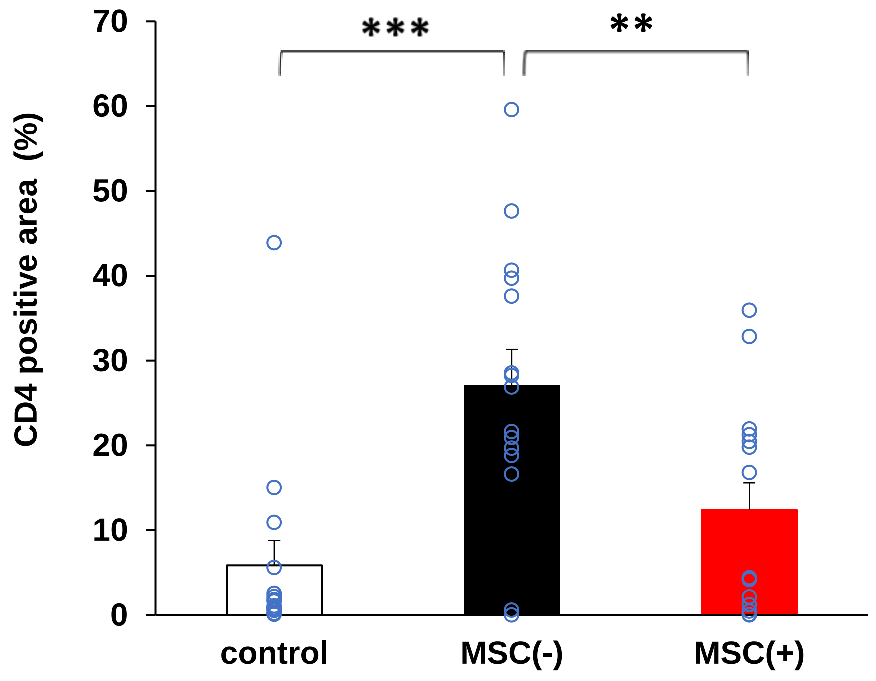

e

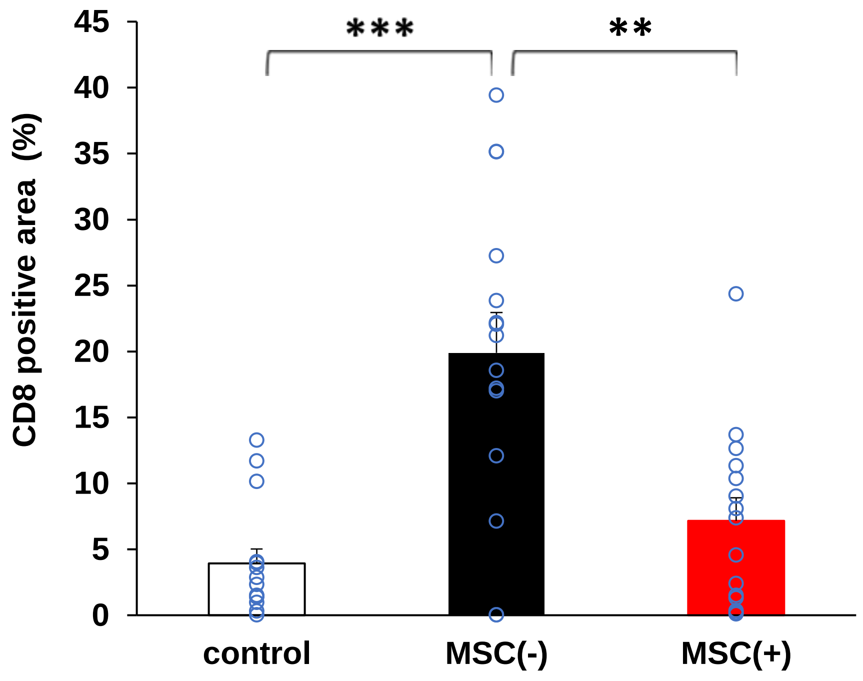

f

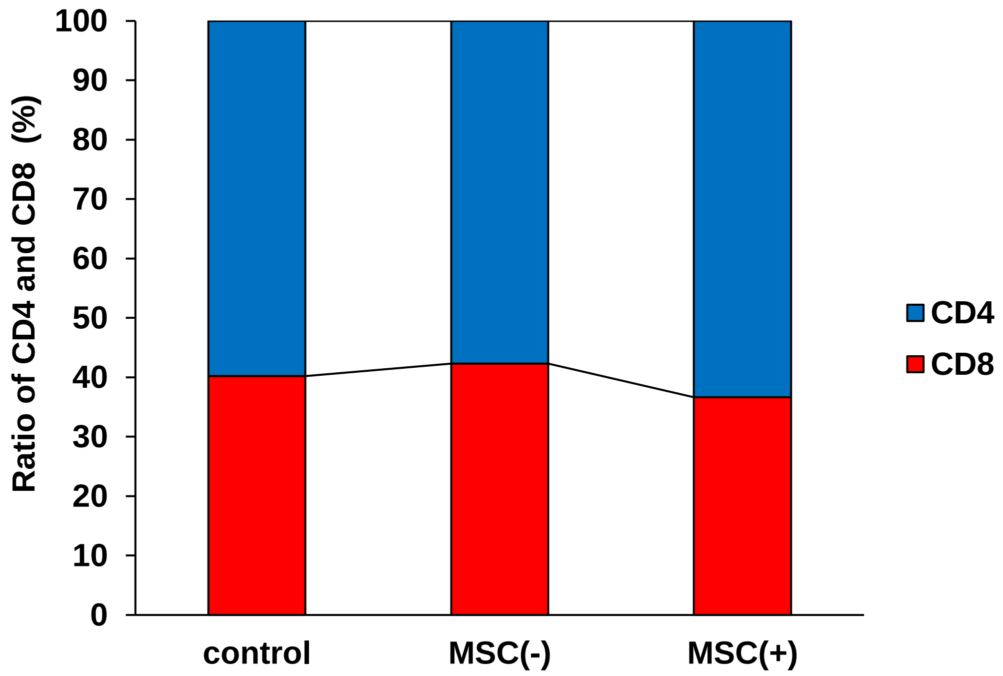

ESM Fig. 3.CD4 and CD8 infiltrations in the islets. (a) Pancreas from a mouse not receiving anti-PD-L1 mAb or hMSCs (control: normoglycemic, insulin content was 2.17 pmol/mg (b) Pancreas from an MSC(-) mouse (diabetic, insulin content was 0.08 pmol/mg). (c) Pancreas from an MSC(+) mouse (normoglycemic, insulin content was 0.66 pmol/mg). (a-c)Each upper panel; Hematoxylin-Eosin staining. Lower left panels; triple immunofluorescence staining for DAPI (light blue), insulin (red), and CD4 (green), and their merge. Lower right panels; triple immunofluorescence staining for DAPI, insulin, and CD8 (green), and their merge. 2 µm serial sections. Scale bars, 100 µm. The pancreatic specimens of the three mice with insulin contents close to the average value in each group were selected as the group representatives. (d) CD4-positive area. (e) CD8-positive area. [control group islets: n=15; MSC(-) group islets: n=15; MSC(+) group islets: n=15]. (f) Ratio of CD4 and CD8 in the same islets [control group islets: n=14; MSC(-) group islets: n=11; MSC(+) group islets: n=15]. Data are shown as the mean ± S.E.M. **p <0.01; ***p <0.001 between the indicated two groups by Student's t-test.

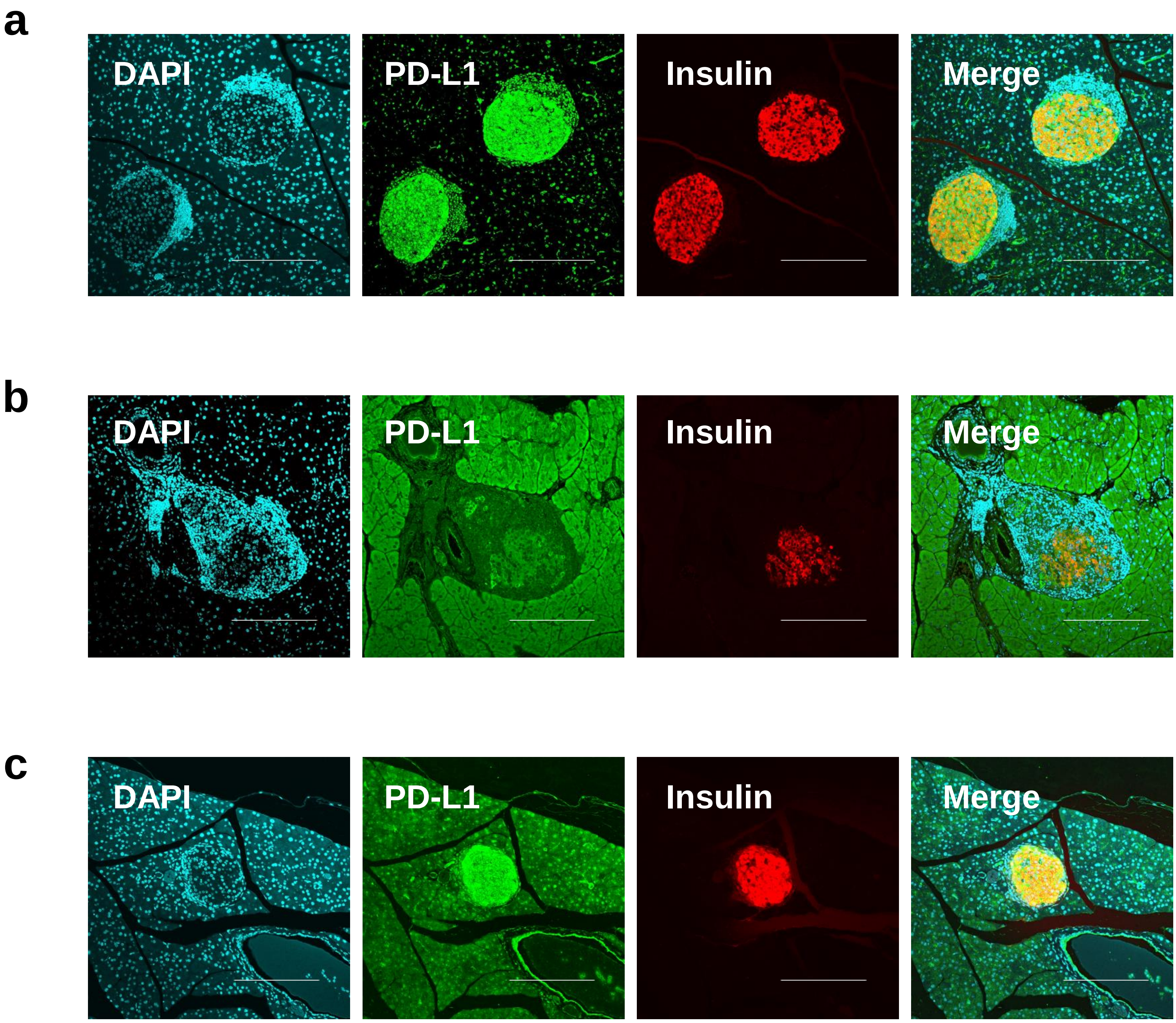

ESM Fig. 4. Representative immunostaining of PD-L1 in the pancreas of NOD mice. Triple immunofluorescence staining for DAPI (light blue), insulin (red), and PD-L1 (green), and their merge (a-c). (a) Pancreas from a mouse not receiving anti-PD-L1 mAb or hMSCs (control: normoglycemic, insulin content was 2.17 pmol/mg). (b) Pancreas from an MSC(-) mouse (normoglycemic, insulin content was 0.41 pmol/mg). (c) Pancreas from an MSC(+) mouse (normoglycemic, insulin content was 1.00 pmol/mg). Scale bars, 100 μm.
